# High-resolution analysis of the varved succession at Crawford lake across the base of the proposed Crawfordian stage and Anthropocene series

**DOI:** 10.1177/20530196251315454

**Published:** 2025-03-31

**Authors:** Francine MG McCarthy, R Timothy Patterson, Carling Walsh, Krysten M Lafond, Brian F Cumming, Andy B Cundy, Karin Hain, Pawel Gaca, Peter Steier, Arnoud Boom, Paul B Hamilton, Michael FJ Pisaric, Martin J Head, Joseph I Boyce, Neil L Rose, Simon D Turner

**Affiliations:** 1 Brock University, Canada; 2 Carleton University, Canada; 3 Queen’s University, Canada; 4 University of Southampton, UK; 5 University of Vienna, Austria; 6 University of Leicester, UK; 7 Canadian Museum of Nature, Canada; 8 McMaster University, Canada; 9 University College London, UK

**Keywords:** Great Acceleration, GSSP, radionuclides, stable isotopes, varves

## Abstract

Four years after the Anthropocene Working Group (AWG) voted to work toward defining the Anthropocene series/epoch with a base in the mid-20th C, the varved sediments of Crawford Lake (Milton, ON, Canada) were selected as the Global boundary Stratotype Section and Point (GSSP) candidate. The initial major rise in activity of ^239 + 240^Pu had been selected as the primary chronostratigraphic marker to define the base of the Anthropocene, but the precise year when this occurred could not be determined from measurements of samples combining multiple varves. Individual varves from freeze cores collected in April 2023 provide annual resolution for bomb radionuclides, allowing the varve age model to be refined, former assignments determined to have been 1 year too old. The increase in ^239 + 240^Pu activities (calculated from atom concentrations of ^239^Pu and ^240^Pu measured using Accelerated Mass Spectrometry) of 0.0031 Bq/g between varves now assigned to 1951 and 1952 is consistent with the onset of thermonuclear weapons testing on November 1, 1952, so the proposed base for the Anthropocene is at the contact between the light- and dark-coloured laminae deposited in 1952 CE (17.5 cm in core CRA23-BC-1F-B). Sharply lower ^239 + 240^Pu and ^137^Cs activities capture the moratorium from November 1958 to September 1961 before rising quickly to peak activities of ^239 + 240^Pu in 1963 CE. Analysis of individual varves with varying amounts of organic matter and inorganic calcite illustrates the influence of lithology on organic proxies, but the upcore trend toward depleted values of δ^15^N through the 20th C reflects increased fossil fuel combustion worldwide. An inflection point in δ^15^N around 1911 CE is attributed the global impact of the Haber-Bosch process and establishment of nearby steel mills, and another in the early 1950s attributed to the Great Acceleration to which the tipping point in the Earth system is attributed.

## Introduction

**Figure 1.**
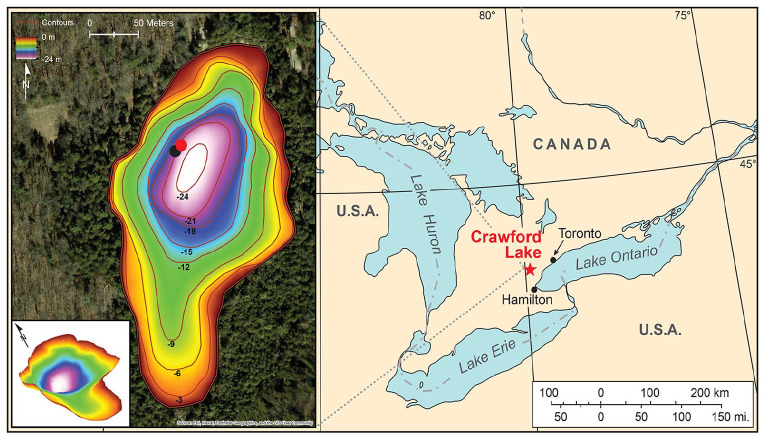
Water below 15.5 m in Crawford Lake (the monimolimnion) is permanently isolated from the mixed layer above (the mixolimnion). Varved sediments accumulate undisturbed by physical or biological agents in the deep basin occupying the northern part of the small lake ~60 km west of Toronto, in the Lake Ontario catchment. Freeze cores were collected from the western part of the deep basin in April 2023. Dots show the location of core CRA23-BC-1F-B (black—the GSSP core proposed by AWG, 2024b) and CRA23- 2FT-A1 (red).

In 2009, the Anthopocene Working Group (AWG) was established by the Subcommission on Quaternary Stratigraphy (SQS) to evaluate the whether the Anthropocene should be introduced as a new unit of the Geological Time Scale, and if so to recommend a Global boundary Stratotype Section and Point (GSSP) to define it. In June 2023, a varved sediment core from meromictic Crawford Lake (43.468658° N, 79.948726° W) near Milton, Ontario, Canada (Figure 1) was chosen by the AWG as the proposed GSSP (AWG, 2024a), which would then define the onset of the Crawfordian Stage and Anthropocene Series as described by McCarthy et al. (2023). This publication had suggested that the varve year 1950 CE serve as the horizon for the GSSP and reported on the plutonium-239 + 240 (^239 + 240^Pu) signal which had become the de facto primary guide to the GSSP (Waters et al., 2023). However, owing to the small amount of material available from thin varves on thin freeze core faces, adjacent varves had to be combined in producing individual analyses; this was particularly an issue with plutonium analysis which required at least 0.6 g dry weight and much of the initial weight of samples (comprising almost entirely authigenic organic matter and calcite crystals) was moisture. Voting by the AWG in June 2023 had, however, left open the question of which year should represent the inception of the Anthropocene, since additional freeze cores obtained in April 2023 had been subsampled at annual resolution for plutonium (^239 + 240^Pu),^137^Cs and organic proxies. Subsequent voting in September to October, 2023 determined that the GSSP horizon should be “at the base of the organic lamina in the varve level equivalent to 1952 CE” (AWG, 2024a), corresponding to the initial major increase in ^239 + 240^Pu that reflects the testing of thermonuclear weapons beginning in November 1952 (Waters et al., 2023).

The results of the coring of sediments from the deep basin of Crawford Lake in April 2023 to fulfil the very precise requirements for an Anthropocene GSSP are discussed below, and the original proposal of McCarthy et al. (2023) updated accordingly with a 1-year adjustment to the original Crawford Lake chronology of Lafond et al. (2023).

In September 2023 the AWG voted to recommend three Standard Auxiliary Boundary Stratotypes (SABSs) to support the proposed Crawford Lake GSSP: cores from Beppu Bay in Japan (Kuwae et al., 2023), Sihailongwan Lake in China (Han et al., 2023), and Śnieżka peatland in Poland (Fiałkiewicz-Kozieł et al., 2023). Key markers of the Nuclear Age and the Great Acceleration can be correlated between these sites and the stratigraphic record described at Crawford Lake, supporting the Anthropocene being distinct from the Holocene.

The refined record of ^239 + 240^Pu and other proxies for the base of the Anthropocene at annual resolution presented in this article bolstered the AWG proposal submitted to the SQS of the International Commission on Stratigraphy (ICS) on October 31, 2023. The boundary between the calcite lamina deposited in the summer of 1952 and the overlying organic lamina that began to accumulate in the meromictic basin of Crawford Lake during fall turnover was proposed as the chronostratigraphic marker to define the base of the Anthropocene. Detonation of the first H-bomb, “Ivy Mike” at Enewetak Atoll in the Marshall Islands at 7:15 AM on November 1, 1952 (2:15 PM on October 31, 1952 in ON, Canada) nominally represents the precise moment associated with the chronostratigraphic boundary, much like the moment of asteroid impact at the end of the Cretaceous nominally marks the onset of the Cenozoic Era.

### The GSSP candidate

Evidence of a substantial globally synchronous shift in the Earth System attributable to human agency was recorded at all 12 sites investigated as potential GSSP candidates to define an Anthropocene epoch/series with support from the *Haus der Kulturen der Welt* (Rosol et al., 2023; Waters et al., 2023). This clear departure from Holocene conditions is associated with the Great Acceleration (Head et al., 2022; Steffen et al., 2015), driven by multiple factors including anthropogenic emissions of CO_2_ that are likely to influence climate for at least 50,000 years—that is, having long-term (“permanent”) impacts that will probably override Milankovitch forcing already suppressed by low orbital eccentricity (Talento and Ganopolski, 2021). Sharply increased concentrations of spheroidal carbonaceous particles/fly ash (SCPs) and depleted values of δ^15^N are evidence of accelerated industrial fuel consumption during the latter half of the 20th century that were measured in multiple records examined by the Anthropocene Working Group (Waters et al., 2023).

**Figure 2.**
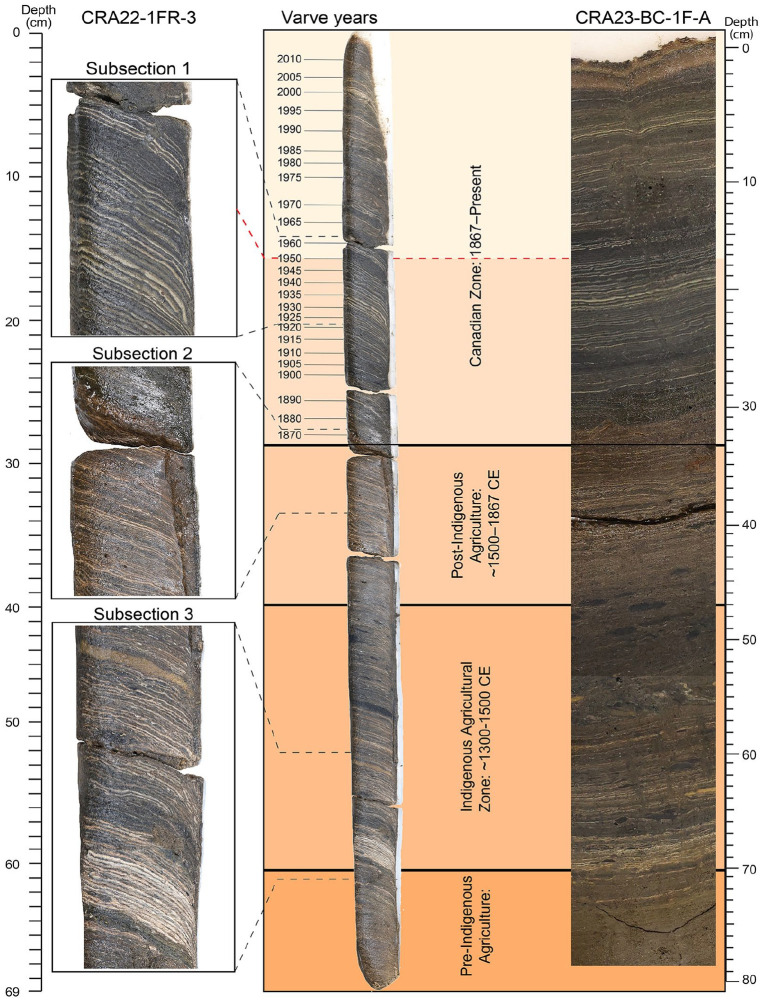
Distinct varves can easily be correlated between the cross section (side-view) of core CRA22-1FR3 (imaged at ultra-high resolution; see Lafond et al., 2023) and the photograph of the recently collected face of core CRA23-BC-1F-A. Distinct varves characterise intervals of anthropogenic impact and cultural eutrophication (Indigenous agricultural settlement in the late 13th through 15th C and colonial impact beginning in the early 19th C), illustrating the difference between the many clear instances of local human impact through the Holocene and the concept of the proposed Anthropocene epoch which reflects the globally synchronous Earth System changes resulting from the Great Acceleration and marked by the first major increase in plutonium activity (AWG 2024b).

Changes in a variety of proxies including myriad novel substances recording a rapidly growing technosphere (Haff, 2014, 2023) and fossil evidence of a transformed biosphere (Williams et al., 2022, 2024) record the effects of the Great Acceleration and the Cold War, distinct from earlier local anthropogenic impacts (Williams et al., 2016). Sharp changes in ‘Anthropocene proxies’ are recorded in the upper metre of varved sediments accumulated below the chemocline of Crawford Lake (Figure S1) that were initially measured on five cores collected from the deep basin in 2019, two collected in 2022, and one freeze core face in storage since 2011 (McCarthy et al., 2023). Subsampling the thin varves (averaging ~1.4 mm through the 20th century; Lafond et al., 2023) from frozen core faces is labour-intensive. Sampling from the base of each light-coloured calcite-rich lamina to the top of the overlying dark-coloured organic-rich lamina requires meticulous attention, and the core faces become increasingly thin upcore. As a result, earlier analyses were conducted on samples combining multiple varve years, to achieve the dry weights required for plutonium analysis using standard counting techniques, representing 2–6 varve years (Table 1). Fortunately, however, the varve pattern is distinctive (Figure 2), allowing confident assignment to coeval varves across cores (see Supplemental Information).

**Table 1.** Results of plutonium analysis from McCarthy et al. (2023), with varve age model following Lafond et al. (2023). The single face corer CRA22-1FR-3 (bold) is only 13.5 cm wide, and a section was retained for archiving as the proposed GSSP candidate, so 4–6 varves were combined for analysis, compared to the 2–4 varve years analysed from the wider cores obtained in 2019 and completely consumed for analysis. Grey shading identifies the first major increase in activity in a sample combining varves deposited in 1950–1953, following background values in the samples deposited in 1948–1951 CE. Sampling failed to capture the bomb peak in 1963, but several samples measure the decline following ratification of the Limited Test Ban Treaty.

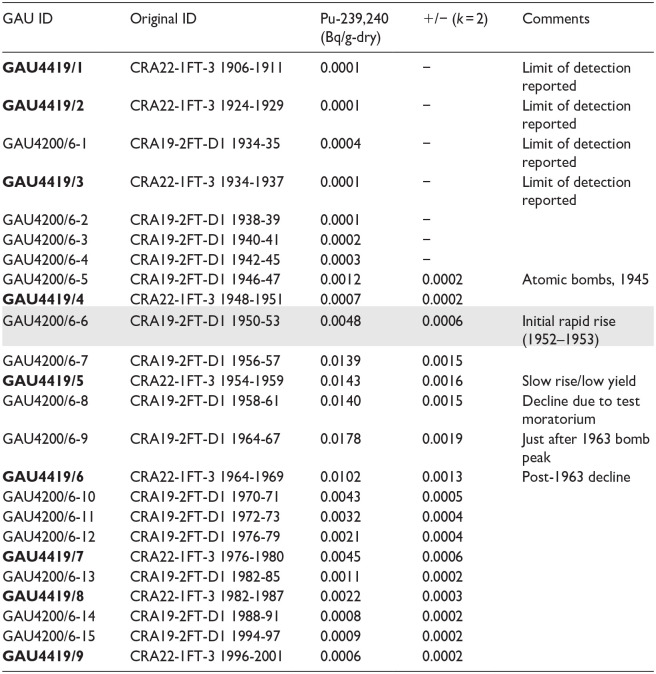				

**Figure 3.**
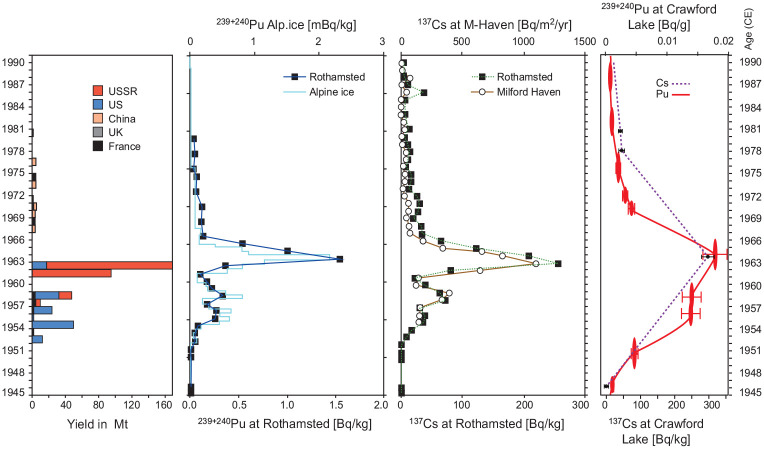
Bomb radionuclide activities measured in an annual herbage archive from Rothamsted, UK, in cores from the Mont Blanc glacier, Milford Haven and Crawford Lake mirror the annual yields of atmospheric fallout from thermonuclear weapons testing (UNSCEAR, 2000). Note the uneven rise to the 1963–1964 peak in both ^239 + 240^Pu and ^137^Cs and clear response to the moratorium between late 1958 and 1961 CE. The earlier increase in radionuclides in varved sediments from Crawford Lake is attributed to proximity to atomic bomb testing in the SW USA. Modified from Warneke et al. (2002), adding data from freeze cores collected from Crawford Lake in 2019 and 2022 from McCarthy et al. (2023).

**Figure 4.**
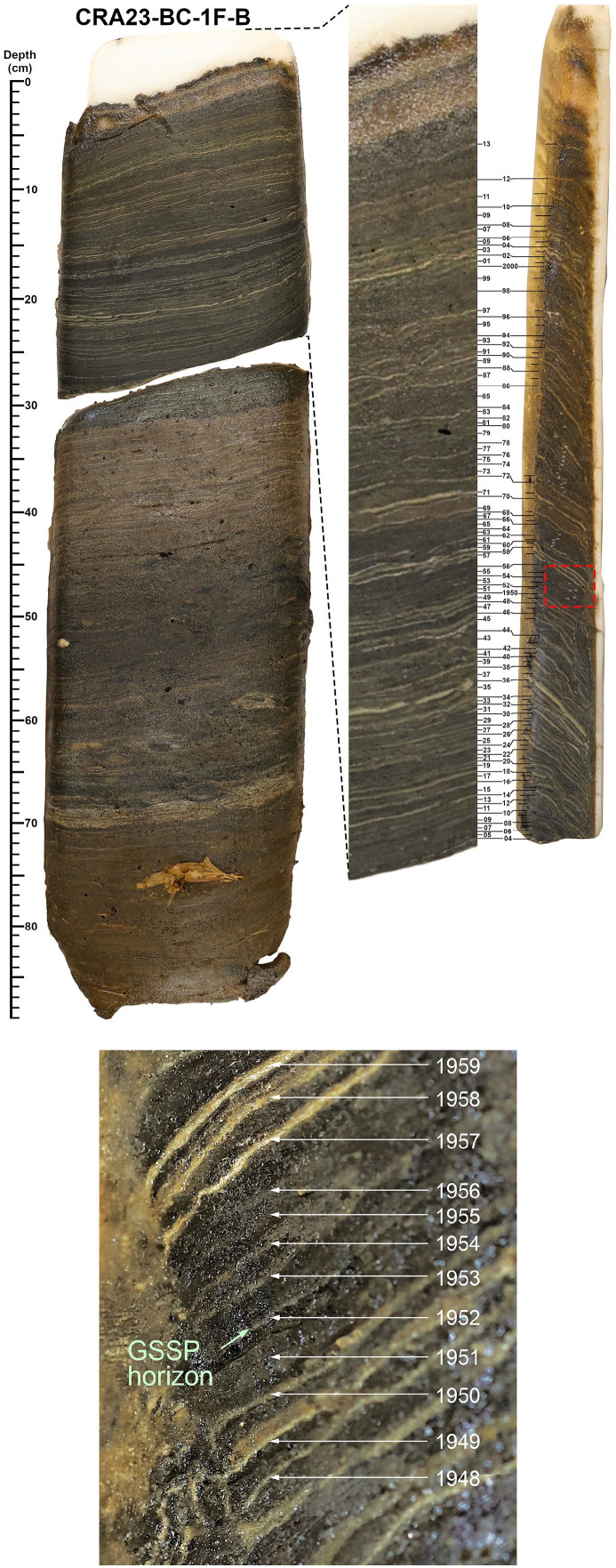
The proposed GSSP is at 17.5 cm in the 89 cm-long freeze core CRA23-BC-1F-B, measured 12.4 cm from the left edge of the core face that is archived at the Canadian Museum of Nature. It is placed at the base of the dark lamina comprised primarily of authigenic organic matter deposited during turnover of the mixolimnion in fall of 1952 CE, and which overlies the thin calcite lamina deposited in the summer. This lithological boundary approximates (and is taken to represent) the moment when the first thermonuclear weapon (Ivy Mike) was detonated: 7:15 AM on November 1, 1952 MHT in the Marshall Islands. This varve is one of several with very thin summer laminae forming the lowermost of two dark bands that are easily correlated between cores across the deep basin of Crawford Lake. The thick calcite lamina at 20 cm, that serves as the starting point for the varve chronology upcore and downcore illustrated at right, is now assigned a calendar age of 1936 CE. Upper panel (left to right): core face, enlargement of core face, edge of core with image reversed to facilitate detailed correlation with the core face. Lower panel: enlarged image of the GSSP interval as indicated by the dashed red box in the upper panel.

The AWG selected ^239^Pu as the primary chronostratigraphic marker for the base of the proposed epoch, since global production and fallout trends of this artificial radionuclide are well-defined, and fallout was synchronous across the globe (UNSCEAR, 2000). Despite its location far from the testing grounds, ^239 + 240^Pu activities measured in the varved sediments of Crawford Lake collected in 2019 and 2022 (red and black symbols in Figure S1, respectively) reflect variations in global yields of plutonium from aboveground testing of thermonuclear weapons (Figure 4), with a decline in activity in the sample dated 1958–1961 attributed to the moratorium that spanned November 1958 to September 1961 CE. The Limited Test Ban Treaty reflected in the steady decline of ^239 + 240^Pu activity is also recorded by the decline in ^137^Cs following peak activity recorded in 1964–1965, albeit with low resolution sampling (Figure S1). The pattern in plutonium activity measured in the varved sediments of Crawford Lake mirrors other records from material (and sites) where plutonium is not readily mobilised (Figure 3). Peak activities measured in the cores recovered from Crawford Lake in 2019 and 2022 were lower than at the auxiliary stratotypes selected to support the proposed GSSP, particularly in the proposed SABS cores from Beppu Bay (Kuwae et al., 2023) and Shihailongwan Lake (Han et al., 2023), but they were comparable to those measured in cores from the Śnieżka peatland (Fiałkiewicz-Kozieł et al., 2023).

The increase from near-limit of detection activities of ^239 + 240^Pu (averaging ~0.0004 Bq/g dry weight) from the 1930s to 0.0048 **± **0.0002 Bq/g in the sample assigned to 1950–1953 using the varve chronology of Lafond et al. (2023) records the onset of thermonuclear testing in late 1952 CE. However, the precise varve year marking this event could not be determined without analysis at annual resolution (Table 1). McCarthy et al. (2023) proposed 1950 CE as the basal age, primarily because this is year 0 BP by convention in radiocarbon dating, but also because this year is already commonly used by the Earth System Science community where the concept that the planetary system had departed from Holocene norms originated. The base of the calcite lamina assigned to 1950 CE using the varve chronology of Lafond et al (2023), at 15.6 cm at the left edge of the archived section of core CRA22-1FR-3 (Figure 2), was proposed as the precise level to define the base of the Anthropocene series/epoch. This horizon is near the base of the lowermost dark band and can easily be correlated between cores and across the deep basin of Crawford Lake.

Sampling resolution was especially low across the proposed base of the Anthropocene in core CRA22-1FR-3 collected in February 2022, archived at the National Biodiversity Cryobank of Canada (Canadian Museum of Nature, Ottawa). To demonstrate the suitability of this freeze core as a GSSP candidate, various key analyses were conducted while also archiving sufficient required material. The rise in ^239 + 240^Pu activity from 0.0007 to 0.0143 Bq/g dry weight between samples dated 1948–1951 and 1954–1959 CE and the sharp increase to over 18,000 SCPs/g dry weight (a 12-fold increase over the early 20th C average) in the sample dated 1952–1953 CE were consistent with measurements in cores collected in 2019: CRA19-2FT-D1 and CRA19-2FT-B2 (Figure S1). High activities of ^137^Cs in the varves dated 1964–1965 CE in the proposed GSSP core record the peak in ‘bomb fallout’ immediately following ratification of the Limited Test Ban Treaty (Figure S1), which was previously only measured in a gravity core from the deep basin of Crawford Lake and subsampled at 1-cm intervals in the field (McCarthy et al., 2023). Additionally, as at other sites studied by the AWG (Hajdas et al., 2023) and in freeze cores recovered from the deep basin of Crawford Lake in 2011 and 2019 (McCarthy et al., 2023), radiocarbon artificially produced by bombardment (the ‘bomb pulse’) was recorded by F^14^C > 1 measured in most sediments deposited in the latter half of the 20th C in spite of the old carbon error measured in the dissolved and particulate constituents of the water column in this karstic basin in 430-million-year-old carbonates of the Lockport Group.

To procure sufficient material to analyse for ^239 + 240^Pu and other key markers at annual resolution across the proposed base of the Anthropocene, and thus determine the precise year when the ‘initial major rise’ in the anthropogenic isotope ^239^Pu occurred, since this was chosen as the primary marker (AWG, 2024a) permission was obtained from Conservation Halton (with the sanction of Indigenous stakeholders) to collect additional freeze cores from the deep meromictic basin in April 2023. This increased resolution is especially critical following the AWG’s decision in October 2023 to propose the varve corresponding to 1952 CE for the GSSP level.

## Materials and methods

### Crawford Lake

Crawford Lake (43.468658° N, 79.948726° W) occupies a sinkhole dissolved in limestones of the Silurian Lockport Group that form the caprock of the Niagara Escarpment (Figure 1). The unique hydrologic conditions promoting the undisturbed accumulation of seasonally controlled authigenic calcite and organic matter in this lake are well understood (Dickman, 1979, 1985; Rybak and Dickman, 1988) despite the unexpected discovery of well-oxygenated bottom waters (Llew-Williams, 2022; Llew-Williams et al., 2024). The lake and its catchment, within a UNESCO World Biosphere Reserve in a Greenbelt region of Ontario, Canada have been protected and managed by Conservation Halton since 1969; yet the lake is within easy reach of international airports in Toronto and Hamilton, Ontario, and Buffalo, New York. Displays at the Interpretive Centre and along an interpretive boardwalk around the lake explain the unique hydrology of the lake, its geologic formation, and potential significance of its varved sediments to Earth history. The reconstructed longhouses onsite attest to the long history of anthropogenic impact on the lake predating European colonization, and an advisory council of Indigenous stakeholders provides input regarding activities in the Crawford Lake Conservation Area, including access to the lake and its associated sedimentary record.

Ease of access and protection of the lake within the Crawford Lake Conservation Area and of the proposed GSSP and parastratotype cores in cryogenic storage at the Canadian Museum of Nature, Ottawa and the Royal Ontario Museum, Toronto were key considerations in the selection of its varved sediments as best representing the departure of conditions from Holocene norms in the mid-20th century). Educational and outreach facilities at the museums and the conservation area provide opportunities to discuss and debate the evidence for large-scale changes to the Earth system worthy of inclusion within the Geologic Time Scale, as well as the prospective societal impacts wrought by these changes worldwide.

The most important consideration was the potential for accurate and precise chronological resolution over the past 2 centuries and a proxy record that can be correlated globally from the proposed GSSP. Crystals of calcite precipitate in the epilimnion of this small (2.4 ha), deep (*Z_max_*. 24 m) hard-water lake each summer and accumulate undisturbed below the chemocline around 15.5 m in the water column, capping predominantly authigenic organic matter (Dickman, 1979, 1985; Llew-Williams et al., 2024). Light-coloured summer calcite layers began accumulating during the late 13th C (Figure 2) when agricultural activity was recorded by a variety of proxies including high concentrations of microscopic charcoal of grasses and other herbs, as well as pollen of cultigens and their fungal pathogens, especially concentrated in goose dung pellets (McAndrews and Boyko-Diakonow, 1989; Byrne and Finlayson, 1998; McAndrews and Turton, 2007, 2010), and confirmed by archeological excavations (Finlayson and Byrne, 1975; Finlayson, 1998). Resulting eutrophication caused the pH of the epilimnion to increase, as carbon is fixed during algal blooms recorded by siliceous (Ekdahl et al., 2004, 2007) and organic-walled (Krueger and McCarthy, 2016; McCarthy et al., 2018) microfossils. Calcite accumulated in discernible layers on the lakebed each summer until the village was abandoned (Ekdahl et al., 2004, 2007), after which calcite deposition was irregular through the Little Ice Age (Llew-Williams et al., 2024).

### Core collection and subsampling

Sediments spanning the last millennium were recovered from a raft above the deepest part of the Crawford Lake basin on April 12 and 13, 2023 within a week following the loss of winter ice (Figure S2). Conditions were ideal, with unseasonably warm temperatures and low winds. A two-face freeze corer from Carleton University, Ottawa and a one-face freeze corer from Queen’s University, Kingston were employed and left in the lakebed for 25–30 minutes to obtain the thickest possible core faces. Eight freeze cores capturing the proposed Holocene–Anthropocene boundary were collected (Tables 2, S2).

**Table 2.** Freeze cores recovering proposed Anthropocene sediments in April 2023 and the type of analysis performed on individual varves. Additional field and subsampling details available in Table S1.

Date of collection	Core name	Water depth (m)	Core length (cm)	Subsampled for analysis
2023/04/12, 8AM	CRA23-2FT-A1	18.3	127	C, N and gamma (^137^Cs)
2023/04/12, 8AM	CRA23-2FT-A2	18.3	117.5	Plutonium
2023/04/12, 10AM	CRA23-2FT-B1	18.3	86.5	–
2023/04/12, 10AM	CRA23-2FT-B2	18.3	86.5	–
2023/04/13, 8:30AM	CRA23-BC-1F-A	18.3	89	Plutonium
2023/04/13, 11AM	CRA23-BC-1F-B	18.3	83	Plutonium
2023/04/13, 2:30PM	CRA23-BC-1F-C	19.2	111.5	–
2023/04/13, 4:00PM	CRA23-BC-1F-D	19.8	87	

Freeze core faces (Figure S3) were carefully detached from the metal samplers on shore (Figure S3) and stored in coolers with dry ice prior to transporting to the Patterson Laboratory at Carleton University where they were stored at −22°C (Figures 4, S2). Prior to subsampling, core faces were photographed and ~5 cm-wide vertical sections were cut from each core using a bandsaw to keep for reference and archive. The remaining sections of the core faces were subsampled at annual resolution. Before subsampling, the core was allowed to thaw slightly to allow the core material to soften and be cut with a scalpel blade. Thawing and subsampling was completed inside a walk-in cool room (+4°C) to maximize the ideal subsampling conditions before the cores needed to be refrozen. Due to the nature of the varve surfaces (typically uneven) and the pull-down of the freeze corer upon penetration that causes a downward curvature of the varves close to the surface of the corer (Figure S4), sampling cannot be done using conventional slicing methods (e.g. a microtome; Macumber et al., 2011). Instead, individual varve years were subsampled delicately by following along the surface of a given varve couplet, from the base of the light-coloured to the top of the dark-coloured lamina, with a scalpel and teasing it apart incrementally from the varve above. Sampling was done in the upcore direction due to the curvature of the varves described above.

Except for vertical sections (~5 cm wide) retained from each core face as archive, individual varves were subsampled across four core faces (Figure S2). Core CRA23-2FT-A was sampled for organic proxy and gamma analysis at annual resolution above the colour change near the base of the Canadian Zone. Cores CRA23-2FT-A2, CRA23-BC-1F-A and CRA23-BC-1F-B were sampled for plutonium analysis at annual resolution between 1945 and 1970 CE. After drying and weighing to ensure that sufficient material was available, samples containing individual varves labelled following the varve chronology of Lafond et al. (2023) were shipped to the Boom Laboratory University of Leicester for organic proxy analysis, to the Paleocological Environmental Assessment and Research Laboratory (PEARL) at Queen’s University for gamma analysis, and to the GAU-Radioanalytical Laboratories, University of Southampton for plutonium separation and analysis. Given the small dry mass of each annually resolved sample, plutonium concentration and isotopic ratio determinations were carried out using Accelerator Mass Spectrometry (AMS), as AMS provides a higher detection sensitivity than standard measurement techniques (such as alpha spectrometry and conventional ICP-MS) for long-lived isotopes such as ^239,240^Pu with t_1/2_ (^239^Pu) = 2.411∙10^4^ years and t_1/2_ (^240^Pu) = 6.563∙10^3^ years. Samples were prepared using a modified protocol adapted to the requirements of AMS in Southampton and then shipped to the University of Vienna (Isotope Physics), Austria, for analysis. Differences to the original protocol published in (McCarthy et al., 2023) are described in the following section ‘Laboratory methods’.

### Laboratory methods

Twenty-four individual varve samples and one sample combining two varve years from core CRA23-2FT-A1 were analysed using a germanium well detector to determine the activity of ^137^Cs. Given the small sample size, all counts were done for 160,000 seconds, double the standard counting time. Unfortunately, there was too little material in the samples labeled 1951 and 1963 for analysis, but additional material was sampled from core CRA19-2FT-B1 to allow analysis of the sample assigned to 1963 CE. The resulting gamma spectra were analysed with the MATLAB program ScienTissME (http://www.scientissime.net/software/) using calibration standards (IAEA 285 and 312) and associated corrections for differences in tube height calibrated to these standards.

Twenty-four samples were carefully subsampled along varve boundaries from cores CRA23-BC-1F-A, CRA23-BC-1F-B and CRA23-2FT-A2 and sent to the GAU-Radioanalytical Laboratories, University of Southampton, where they were prepared for plutonium analysis. Sample preparation used a modified protocol from that reported previously for Crawford Lake samples (McCarthy et al., 2023), using HF digestion of ^242^Pu-spiked samples instead of fusion digestion followed by radiochemical purification (ibid.) prior to precipitation with Fe(OH)_3_ which was then calcined onto low-background (pre-nuclear weapons testing era) Fe_2_O_3_. The co-precipitation replaced the electrodeposition step of the original protocol as AMS requires the radionuclide to be embedded in a few mg of solid carrier material. The Fe_2_O_3_ was then pressed into aluminium sample holders for AMS measurement. ^239^Pu and ^240^Pu concentrations were analysed using the Vienna Environmental Research Accelerator (VERA) at the University of Vienna analogous to the samples from the Karlsplatz site in Vienna (Wagreich et al., 2022). The present setup and the measurement routine for actinides such as ^239^Pu and ^240^Pu have been described in detail in (Steier et al., 2019). AMS reaches its exceptional sensitivity by combining several mass spectrometers with a tandem accelerator with which a complete suppression of molecular background is achieved. The successful suppression of uranium hydrides in the present measurement was monitored by analysing mass 239 also on in-house U standards (Vienna KkU (^236^U/^238^U = (6.98 ± 0.32) × 10^−11^ (Steier et al., 2008)) which contains 100 μg of ^238^U but no ^239^Pu, that is, below the detection limit. The absolute number of atoms in the sample is obtained by measuring mass 239 and 240 relative to mass 242 of the added ^242^Pu spike. In that way, losses during chemical sample preparation and the detection efficiency are accounted for, as all three masses are counted in the same final detection system. The three masses are measured sequentially by changing the electric components of the AMS setup. The blank corrected values of ^239^Pu and ^240^Pu were then converted to an activity by their respective half-lives and added up to obtain the sum activity of ^239 + 240^Pu. Blanks were one order of magnitude lower than the lowest sample of the batch in terms of the normalized count rate. The final activities are given as a specific ^239 + 240^Pu activity by normalizing to the dry weight of the sample for comparison with previous analyses by alpha spectrometry on sediments from Crawford Lake and the other sites analysed in the search for a GSSP for the Anthropocene. In this article, we report results across the initial increase in Pu activities due to the onset of high-yield thermonuclear weapons testing in the early 1950s (samples 4–9) and the period preceding and following the ratification of the Limited Test Ban Treaty in 1963 CE (samples 16–21), to discriminate the proposed primary chronostratigraphic marker for the base of the Anthropocene to an annual resolution, and tie-in the previously reported Pu data trends for Crawford Lake to an annual varve chronology (and to annual regional and global fallout trends).

Carbon and nitrogen analysis (including stable isotopes) was conducted on 128 individual varve year samples through the Canadian Zone of core CRA23-2FT-A1. Data were obtained using an elemental analyser (ANCA GSL, Sercon, UK), combusted over chromium (III) oxide at 1000°C and reduced over activated copper at 600°C. Water was removed using a magnesium perchlorate scrubber and N_2_ and CO_2_ were separated using gas chromatography. Isotopic ratios and quantifications were determined using a mass spectrometer (20-20MS, Sercon, UK). Total nitrogen (%N) and carbon (%C) are expressed in mass as percentage values, C:N ratios were calculated on a mass basis, and stable isotopic composition relative to the standards were expressed in permille values using delta notation: δX = [(Rsample/Rstandard) − 1] × 100, where δX is δ^13^C versus V-PDB or δ^15^N versus air, R is the ^13^C/^12^C or ^15^N/^14^N ratio. Most data points represent triplicate analyses, and precision and accuracy were 0.1 permille (‰) for both δ^15^N and δ^13^C.

## Results

### Chronological controls

**Figure 5.**
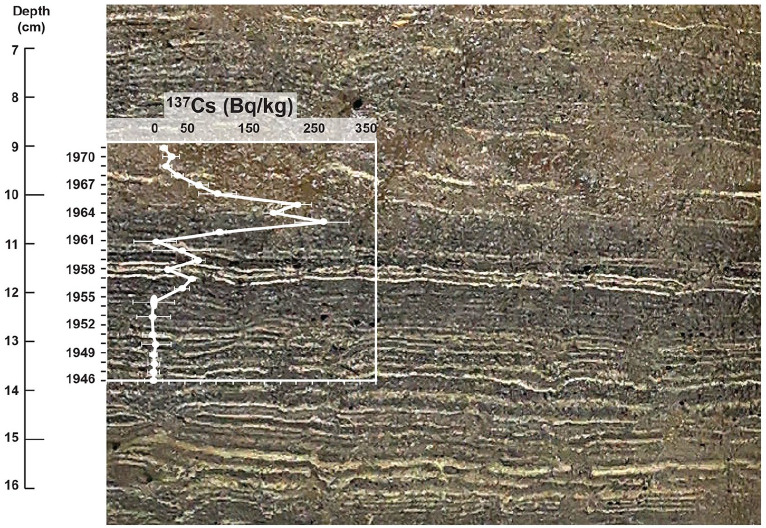
Gamma activity attributed to ^137^Cs in varves from core CRA23-2FT-A is consistent with global fallout records, capturing the 1958–1961 moratorium as well as the decline following ratification of the Limited Test Ban Treaty in late 1963 CE (compare Figure 3). All analyses (spanning less than 5 cm in the core, illustrated here) are of individual varves except 1953–1954 CE, which were combined for analysis, and thus illustrated by an ellipse rather than a circle.

Gamma analysis of individual varves from face 1 of core CRA23-2FT-A (Tables 3, S2, Figure 5) measured initial ^137^Cs activity of 44.1 ± 11.6 Bq/kg in the varve assigned to 1955 CE based on the age model of Lafond et al. (2023) that attributed the thickest white lamina of the warm, dry ‘Dust Bowl’ interval to 1935 CE. ^137^Cs activity increased unsteadily to 70.2 ± 10.1 Bq/kg in the varve labeled 1958 CE and declined to background values with very large error bars in two samples: 44 ± 45.8 Bq/kg in 1959 and 2.5 ± 34.3 Bq/kg in 1960 CE. Sharply higher activity in the sample labeled 1961 (103.2 ± 10.2 Bq/kg) was followed by peak ^137^Cs activity in the sample assigned to 1962 CE (265.2 ± 42.3 Bq/kg). Activity measured in the 1963 CE sample that combined material from both cores CRA23-2FT-A and CRA19-2FT-B1 was 189.6 ± 16.8 Bq/kg, unexpectedly slightly lower than the overlying sample labeled 1964 (225.3 ± 24.2 Bq/kg), although the error bars suggest that activities could have been approximately equal in both samples. A sharp decline followed to 100.5 ± 30 Bq/kg in the sample labeled 1965, followed by a steady decline to background levels in the sample labeled 1968 (19.4 ± 9 Bq/kg) after which values remained stable within the error of estimate.

**Table 3.** Results of gamma analysis of individual varves in core CRA23-2FT-A, with original varve ages (sample IDs) following the model of Lafond et al. (2023). Shading highlights peak activity in the varves labeled 1962–1964 CE, with revised varve ages at right; note the sharply lower values in the varves deposited during 1959 and 1960 CE, during the nuclear test moratorium.

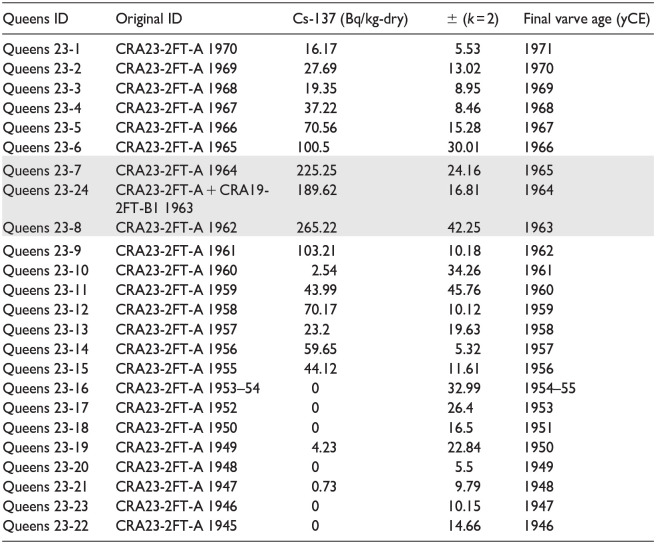				

**Figure 6.**
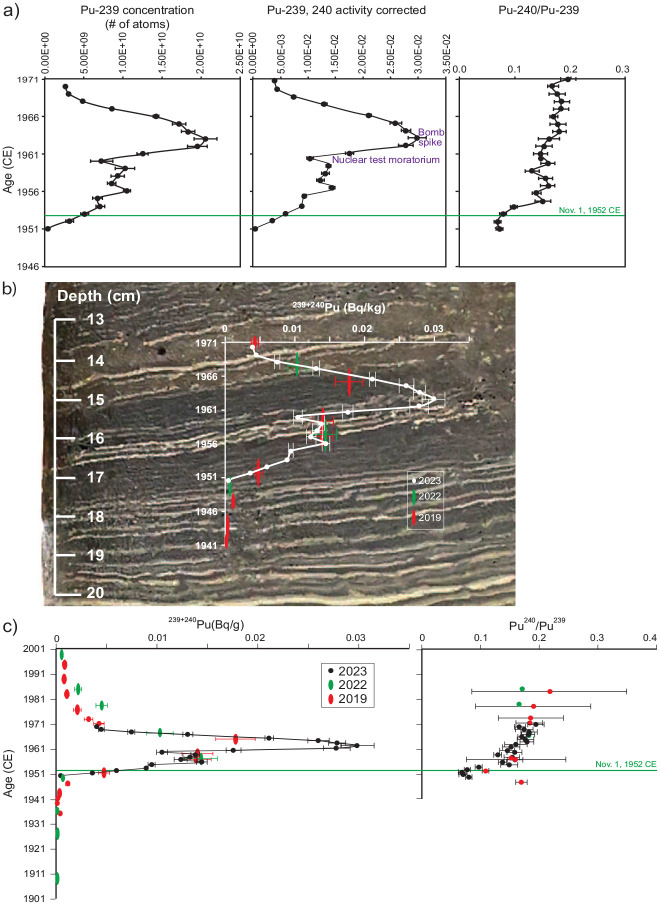
Plutonium isotopes across the proposed base of the Anthropocene (green line, base of the calcite lamina in the varve deposited in 1952 CE) in varved sediments from Crawford Lake. (a) Blank-corrected concentrations of atoms of ^239^Pu per dry weight measured using accelerator mass spectrometry (AMS) in 18 individual varves from freeze cores collected in 2023, activities of ^239^Pu and ^240^Pu calculated from the atomic concentrations, and ratios of ^240/239^Pu (Table 4). (b) Plutonium activities at annual resolution plotted with data from freeze cores collected in 2019 and 2022 (red and green symbols), all subsampled with reference to the same thick, distinctive marker varve visible at lower right in the photograph of the proposed GSSP core CRA23- BC-1F-B. (c) Plutonium activities are clearly consistent with global yields, increasing steadily to 1954 CE from very low activities in 1951 CE, and rising quickly from 1961 to the peak in 1963 CE, more clearly identified with higher resolution sampling of individual varves. The data therefore record both the upturn of Pu activities following the onset of high-yield thermonuclear weapons testing in 1952, and earlier testing activity. The low activities measured in 1961 CE confirm the shoulder in the profile that identifies the 1958–1961 moratorium.

The apparent 1-year offset in peak measurements of ^137^Cs, supported by the peak in plutonium activity in the sample labeled 1962 (Tables 4, S4, Figure 6), led us to reexamine the varve age model by comparing the Canadian Zone (since 1867 CE) in all freeze cores collected from Crawford Lake, including core CRA23-BC-1FR-A that appeared to best capture uncompacted sediments on the lakebed. The distinct thick varve used as a reference for sampling upcore and downcore had been assigned to 1935 CE on the assumption that this was the warmest, driest year of the Dust Bowl, thus producing an exceptionally thick calcite lamina, because the most important variables affecting precipitation of calcite are temperature and pH of the epilimnion (Llew-Williams et al., 2024). However, subsequent investigation of meteorological variables measured in Toronto revealed that the hydrologic/water year 1936 (October 1935 through September 1936) was characterised by a warmer and drier summer than 1935 (mean JJAS temperature = 25.3°C and JJAS precipitation = 211.6 mm in 1936 compared with mean JJAS temperature = 24.2°C and JJAS precipitation = 270.9 mm in 1935 in Toronto, Table 5, S3 (Environment and Climate Change Canada, 2024)). Sediment trap analysis paired with almost monthly measurements of hydrologic conditions quantified the requirements for calcite to precipitate: water temperatures >15°C and pH values (>7.8) that are promoted by primary production in the epilimnion and uppermost metalimnion that sequesters of CO_2_ and low meteoric input, minimizing dilution of ions from the dolomitic limestone bedrock (Figure S5). Thick calcite laminae are thus promoted by hot, dry summers, making 1936 CE the more likely candidate for the thickest light-coloured lamina of the Dust Bowl. Additionally, 1936 followed several very dry and warm summers through the early 1930s, while 1937 CE was an exceptionally wet year (mean JJAS precipitation = 275.2 mm, total annual precipitation 857.2 mm), bringing an end to the peak drought interval in this area, explaining the thinner calcite lamina deposited that year. The connection between temperature and rainfall became complicated due to the onset of substantial acid rain precipitation in the early 1950s. This precipitation stemmed from a significant increase in industrial emissions of sulfur dioxide (SO_2_) and nitrogen oxides (NO_x_) linked with the Great Acceleration, which remained largely unregulated until the enactment of a sequence of clean air acts in Canada and the USA commencing in the early 1970s (Burns et al., 2016). During the 1950s and 1960s, acid rain led to the formation of thin calcite layers, featuring distinguishable dark bands that could be readily matched across cores. Conversely, thicker calcite layers reappeared as acid rain declined, particularly during warm, arid summers from the 1970s onwards. Close analysis of the varve succession in several core faces collected between 2019 and 2023 suggest a slightly greater offset below the marker varve to the colour change in the late 19th C that is more consistent with historic land-use data. The imaging technique outlined in Lafond et al. (2023) was used to capture the profiles of several 2019–2023 cores, allowing for comparison and confirmation of the varve stratigraphy. While the varves in Crawford Lake are generally laterally continuous, their appearance in photographs may vary due to a variety of factors, such as ambient light conditions, the method used for cutting the core, whether flash photography was used, and how thawed the core was during photography. By imaging multiple cores and correlating various distinct varves across each core profile, varves were recognized with greater certainty despite variation in their appearance, allowing the varve succession to be more accurately and precisely aligned than in previous attempts.

**Table 4.** Results of plutonium analysis of individual varves from freeze cores collected in April 2023, with original varve ages (sample IDs) following the model of Lafond et al. (2023). Activity (bold; associated errors in regular font) was calculated from the concentration of atoms of ^239^Pu and ^240^Pu measured using AMS. Shading highlights the initial major rise in ^239+240^Pu activity and lowest ^240/239^Pu ratio in the varve deposited in 1952 CE, the decline attributed to the moratorium in 1960, and the peak in ^239+240^Pu activity in 1963 CE. The lowest ^240/239^Pu values were measured in the sample from 1952 CE, and together with the almost sixfold increase in ^239^Pu, this marks the base of the proposed Anthropocene series/ epoch in the varved succession from Crawford Lake. Note that sample numbers 1–3 and 10–15 are at GAU awaiting preparation for analysis. Final (corrected) varve ages are shown in bold in the final column.

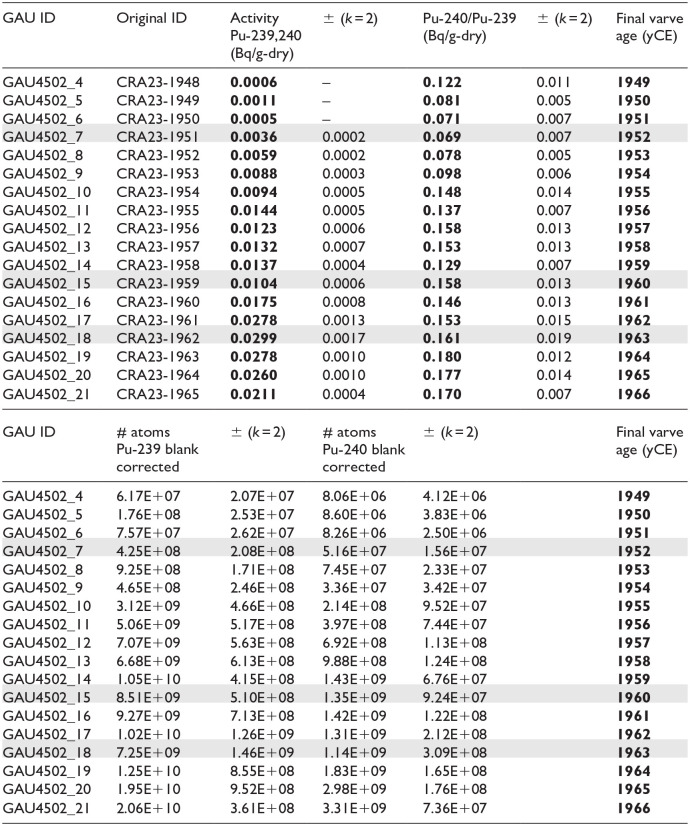						

**Table 5.** Meteorological data from Toronto Station (Climate ID 6158350), 43.6667, -79.4000, elevation 112.5 m. (see Supplementary Table S3 for complete dataset) Summer (June, July, August, September: JJAS) conditions were more conducive to calcite precipitation in 1936 CE, despite the lower total annual precipitation and slightly higher mean annual measured in 1935 CE (grey shading) that was originally interpreted as producing the thickest calcite lamina during the exceptionally arid and warm 1930s. The calcite lamina deposited during the dry summer of 1952 CE and the three distinct white laminae deposited in the relatively dry 1957, 1958 and 1959 are relatively prominent in the lower dark band facilitating correlation across all cores.

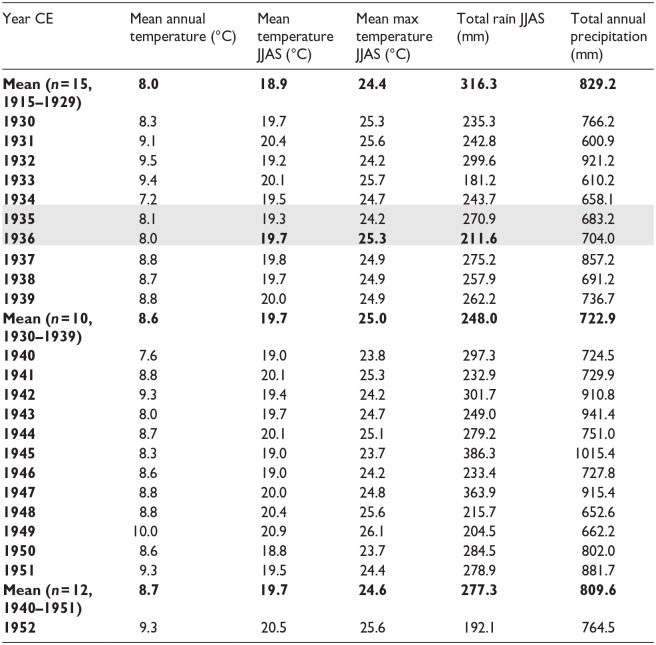					

The varve age model was thus refined, and the results of plutonium and organic proxy analysis of the 2023 cores in this study are depicted against the varve years based on the slightly revised age model. This change entailed adding 1 year to samples above the thick marker varve (now assigned from 1935 to 1936 CE) relative to the model of Lafond et al. (2023) as used in McCarthy et al. (2023). The original labels are clearly indicated in all tables to facilitate comparison with previously published data. The discussion that follows is based on this slight revision to the age model, and comparison with data published earlier takes this refinement of the varve chronology into account.

### Anthropocene markers

Accelerator mass spectrometry of 18 individual varves (Table 4) assigned to 1949–1966 CE using the updated chronology captures the initial consistent increase in fallout from testing of thermonuclear weapons beginning in 1952 CE to the Cold War peak in 1963 CE (Figure 6). A steady increase in ^239^Pu concentration (blank corrected per g dry mass with 1 sigma uncertainty and calculated alpha activity, ^239 + 240^Pu Bq/g, blank corrected per g dry mass) from pre-Cold War levels in 1951 CE (4.65 ± 0.26)∙10^8^ atoms/g (0.0005 Bq/g) was measured from 1952 (3.12 ± 0.21)∙10^9^ atoms/g; 0.0036 ± 0.0002 Bq/g) to 1954 CE (7.07 ± 0.25)∙10^9^ atoms/g; 0.0088 ± 0.0003 Bq/g). Values of the ^240^Pu/^239^Pu atom ratio are low between 1949 and 1954 CE, ranging from 0.122 ± 0.011 in 1949 to the lowest ratios measured, 0.069 ± 0.007 in 1952 CE (Table 4). Values of ^240^Pu/^239^Pu are much higher in varves deposited in 1955–1966 CE, ranging from 0.129 ± 0.007 in 1958 to 0.180 ± 0.012 in 1964 CE. Calculated ^239 + 240^Pu activity increased quickly from 0.0094 ± 0.0005 in 1955 to 0.0144 ± 0.0005 Bq/g in 1956 and remained relatively stable until 1960 when they declined to 0.0104 ± 0.0007 Bq/g. Activity subsequently rose quickly to the peak 0.0299 ± 0.0017 Bq/g in the varve assigned to 1963 CE where the highest concentration of ^239^Pu (20.6 ± 1.5)∙10^9^ atoms/g was measured in the varve assigned to 1963 CE. Concentrations and activities decline steadily upcore to (14.2 ± 0.6)∙10^9^ atoms/g and 0.0211 ± 0.0004 Bq/g in 1966 CE.

### Organic proxies

**Figure 7.**
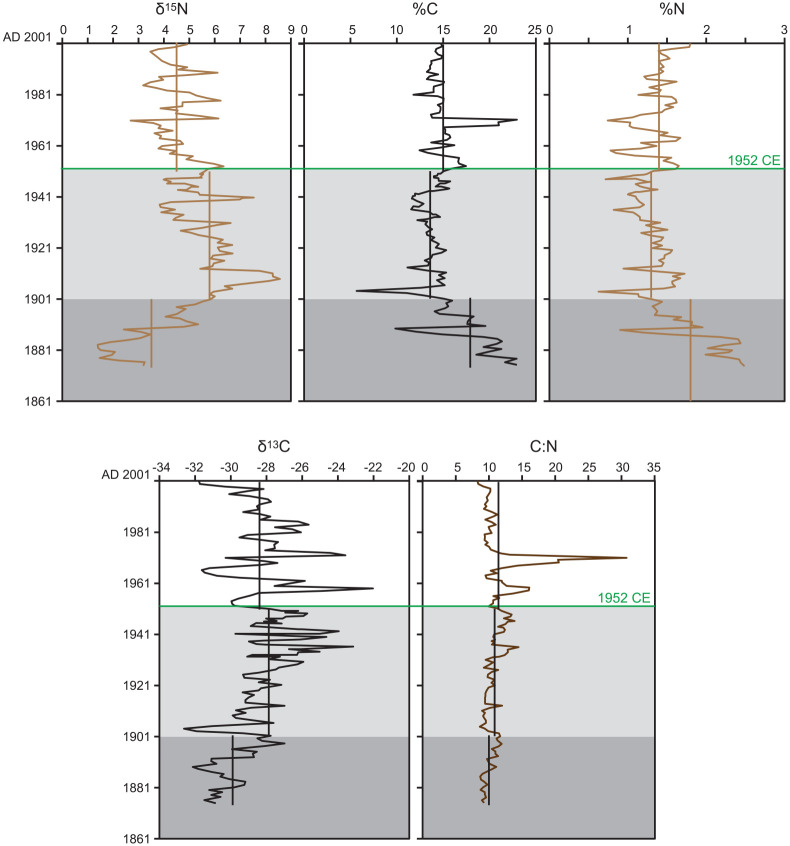
Carbon and nitrogen records for core CRA23-2FT-A1. Generally, C and N covary in endogenic varves composed almost entirely of authigenic calcite capping organic matter derived primarily from algae and their consumers, so C:N ratios tend to hover around the mean until the mid-20th C, when very high C:N ratios and less depleted values of δ13C were measured in varves with prominent calcite laminae. Vertical lines illustrate mean values of organic proxies in individual varves during the late 19th C (dark grey shading), early 20th C (light grey) and late 20th C (no shading). Stable isotopes were most depleted in dark calcite-poor, highly organic sediments (mean %C = 17.9%, %N = 1.8%) deposited in the late 19th C, when a lumber mill operated on the south shore of the lake. The proposed Anthropocene epoch (above the green line, 1952 CE) is characterised by depleted values of δ15N (mean ~1.3 ‰ lower than during the first half of the 20th C), although the overall trend is toward increasingly depleted values upcore since 1912 CE.

As with all other cores from the deep basin of the meromictic Crawford Lake, cores collected in April 2023 show a marked color change from reddish brown sediments to very dark brown organic matter (Figure S2), attributed to intense logging and establishment of a lumber mill on the south shore of Crawford Lake (McCarthy et al., 2023). This ‘Canadian Zone’ was analysed at annual resolution in 128 samples deposited between 1875 and 2001 CE. Total nitrogen (%N) values were low, 0.6%–2.5% (mean = 1.4%), with highest values in dark sediments just above the colour change dated to 1879 CE, except for a few intervals (typically supported by multiple varve years in 1887–1889 and 1902–1904 CE) when %C was also low, suggesting inorganic/ terrigenous mineral influx (Figure 7, Table 6). Following a gradual decrease in nitrogen and carbon, that reached lowest values (%N = 0.6, %C = 5.8) in 1904 CE, values remained relatively stable about the mean through the remainder of the early 20th C (mean %N = 1.3%, %C = 13.6%) and C/N values hover around the early 20th C mean of 10.8. Considerable variability is associated with relative influx of authigenic calcite and organic matter, with strong peaks in 1936, 1957–1959, and 1968–1971 associated with prominent light-coloured laminae, and the strongest peak in the exceptionally thick (2.6 mm) varve dating to 1971 CE, with thick dark and light laminae (see varve thicknesses in Lafond et al., 2023). Perhaps surprisingly, despite the clear abundance of inorganic carbon in the prominent (although thin) calcite laminae, %C values measured in samples from 1957, 1958 and 1959 CE were slightly below the mean for the late 20th century of 15% C, ranging from 12.5%–14.8%, suggesting that the organic carbon fraction of these varves was low, so mineral content must have been slightly elevated. Varves spanning 1969–1971 CE are also exceptionally nitrogen-poor (0.7–1 %N) and are characterized by highly depleted δ^15^N values (2.7–3.9 ‰).

**Table 6.** Results of organic proxy analysis of individual varves in core CRA23-2FT-A. Shading highlights peak activity in the varves labeled 1962–1964 CE. The revised varve age model adds 1 year to each sample collected using the chronology of Lafond et al. (2023) throughout, which is accurate for sediments younger than 1936 CE. Age offset to the late 19th C colour change (re-assessed to 1879 CE following a more comprehensive assessment of varve chronology across all cores) will require more careful reassignment but since this does not affect the base of the proposed Anthropocene, the affected organic proxy data are identified using grey shading here. Bold identifies anomalous samples, and these typically correlated with changes in inorganic elemental and biotic markers.

Age (CE)	%N	^15^N	%C	^13^C	C:N
1875	2.49	3.23	22.93	−30.86	9.2
1876	2.44	3.26	21.77	−31.48	8.9
1877	2.41	2.26	22.83	−30.59	9.5
1878	2.26	1.44	21.12	−31.10	9.3
**1879**	**1.99**	**1.99**	**18.66**	**−30.48**	**9.4**
1880	2.29	2.05	20.02	−31.21	8.7
1881	2.34	1.53	21.29	−29.82	9.1
1882	1.99	1.44	19.33	−29.24	9.7
1883	2.23	1.42	20.81	−29.22	9.3
1884	2.43	1.90	21.27	−30.06	8.8
1885	2.41	2.55	20.84	−30.57	8.7
1886	2.17	3.07	19.31	−30.42	8.9
**1887**	**1.53**	**3.48**	**14.90**	**−31.04**	**9.7**
**1888**	**1.17**	**3.12**	**12.09**	**−31.79**	**10.4**
**1889**	**0.89**	**2.43**	**9.92**	**−32.09**	**11.1**
1900	1.95	4.78	19.58	−30.80	10.1
1901	1.30	5.84	15.12	−27.76	11.7
1902	1.13	5.97	13.05	−29.34	11.5
**1903**	**1.12**	**5.83**	**11.08**	**−31.91**	**9.9**
**1904**	**0.62**	**5.97**	**5.78**	**−32.62**	**9.3**
1905	1.46	6.69	12.53	−31.00	8.6
1906	1.59	6.42	15.08	−27.60	9.5
1907	1.56	7.07	14.80	−28.71	9.5
1908	1.56	8.12	14.44	−29.68	9.2
1909	1.67	8.57	15.11	−29.89	9.1
1910	1.57	8.28	14.64	−29.16	9.3
1911	1.71	8.30	15.24	−29.65	8.9
1912	1.33	7.75	13.36	−28.86	10.1
**1913**	**0.94**	**5.44**	**11.19**	**−26.93**	**12.0**
1914	1.43	5.93	13.40	−29.18	9.4
1915	1.45	5.97	13.48	−29.13	9.3
1916	1.38	6.37	13.04	−29.04	9.4
1917	1.46	5.86	13.75	−28.74	9.4
1918	1.46	5.94	13.84	−29.31	9.5
1919	1.54	6.71	14.85	−28.43	9.6
1920	1.55	6.21	15.35	−28.26	9.9
1921	1.31	6.12	14.22	−27.18	10.9
1922	1.42	6.70	14.69	−28.42	10.3
1923	1.315	6.12	14.206	−27.81	10.8
1924	1.41	6.30	13.74	−29.22	9.7
1925	1.44	5.89	14.03	−29.30	9.9
1926	1.28	5.38	13.30	−28.41	10.4
1927	1.16	5.05	13.20	−27.50	11.4
1928	1.50	4.68	13.85	−27.29	9.3
1929	1.35	5.48	13.43	−26.27	9.9
1930	1.22	6.12	13.12	−25.99	10.8
1931	1.41	6.63	13.29	−27.83	9.4
1932	1.15	4.40	13.30	−27.26	11.5
1933	1.15	4.50	14.09	−26.27	12.3
1934	1.10	4.78	14.19	−25.01	12.9
1935	1.06	3.90	13.64	−26.10	12.9
**1936**	**0.81**	**4.42**	**11.91**	**−23.13**	**14.7**
1937	1.14	3.91	12.35	−28.09	10.9
1938	1.20	3.87	12.71	−28.14	10.6
1939	1.14	4.30	12.52	−26.08	11.0
1940	1.10	6.99	11.83	−25.99	10.7
1941	1.09	7.12	11.99	−29.70	11.0
1942	1.00	5.44	12.41	−23.97	12.4
1943	1.13	5.19	13.95	−26.20	12.3
1944	1.36	4.88	15.72	−28.31	11.5
1945	1.15	5.35	14.27	−27.12	12.4
1946	1.10	5.00	15.13	−27.37	13.8
1947	1.28	4.28	15.78	−28.02	12.3
1948	1.09	4.12	14.53	−25.92	13.4
1949	1.04	5.47	14.35	−25.71	13.76
1950	1.19	5.55	14.70	−26.26	12.34
1951	1.33	5.60	15.08	−27.80	11.3
1952	1.61	5.74	15.91	−29.74	9.9
1953	1.6	6.35	17.39	−29.94	10.6
1954	1.57	5.95	16.73	−29.96	10.6
1955	1.45	5.35	16.60	−29.58	11.4
1956	1.55	4.89	16.64	−28.87	10.8
1957	1.09	5.12	14.82	−28.42	13.6
**1958**	**0.84**	**4.23**	**13.41**	**−24.84**	**15.9**
**1959**	**0.78**	**4.49**	**12.47**	**−22.02**	**16.0**
1960	1.16	3.80	14.60	−27.502	12.6
1961	1.34	3.91	16.23	−30.30	11.9
1962	1.16	4.73	13.72	−25.84	11.9
1963	1.61	4.65	15.47	−29.48	9.5
1964	1.66	3.86	15.79	−30.77	9.6
1965	1.40	3.88	15.68	−31.45	11.2
1966	1.49	3.64	15.26	−31.60	10.2
1967	1.28	4.34	15.28	−31.19	12.0
1968	1.05	3.81	15.13	−28.36	14.4
**1969**	**1.03**	**3.87**	**21.02**	**−27.38**	**20.5**
**1970**	**1.03**	**3.60**	**21.00**	**−28.15**	**20.4**
**1971**	**0.75**	**2.70**	**22.99**	**−30.28**	**30.7**
1972	1.06	6.14	13.87	−23.57	13.1
1973	1.16	5.43	13.69	−24.42	11.8
1974	1.43	4.49	14.58	−28.07	10.2
1975	1.47	4.55	14.74	−27.53	10.0
1976	1.56	3.89	14.75	−27.52	9.4
1977	1.47	4.74	14.42	−27.37	9.8
1978	1.62	4.74	15.03	−28.49	9.3
1979	1.59	6.23	15.01	−29.51	9.4
1980	1.55	5.72	14.48	−29.07	9.3
**1981**	**1.13**	**5.27**	**11.81**	**−26.03**	**10.4**
1982	1.39	5.01	13.98	−26.55	10.1
1983	1.42	4.12	14.01	−27.50	9.9
1984	1.27	3.69	13.97	−25.63	11.0
1985	1.43	3.19	14.96	−26.24	10.4
1986	1.61	3.47	15.13	−28.32	9.4
1987	1.24	3.98	13.26	−27.78	10.7
1988	1.21	3.84	13.51	−28.38	11.2
1989	1.40	4.85	14.51	−29.34	10.4
1990	1.44	6.12	13.26	−28.40	9.2
1991	1.40	4.60	13.60	−28.50	9.7
1992	1.45	4.92	13.56	−28.38	9.4
1993	1.40	4.27	13.77	−27.74	9.8
1994	1.39	4.01	13.65	−27.95	9.8
1995	1.53	3.87	14.75	−28.97	9.7
1996	1.49	3.76	14.83	−30.09	10.0
1997	1.42	3.66	14.49	−28.96	10.2
1998	1.40	3.50	14.20	−28.20	10.2
1999	1.56	3.79	14.24	−30.35	9.1
2000	1.78	4.42	14.92	−31.73	8.4

The most depleted values of δ^15^N were measured in varves deposited during the late 19^th^ C, with a mean ~3.5‰ (*n* = 26) and the lowest value measured throughout the 128-year dataset was 1.4‰, in the varve labeled 1882 CE. The most enriched δ^15^N values (>8‰) were measured in samples from the early 1900s, and there is a declining trend in δ^15^N beginning in 1912 CE, with the lowest values from the mid-1950s through 1970s, where the most depleted value of the 20th century was measured, 2.7‰ in the varve deposited in 1971. The distinct triplet of light-coloured laminae deposited in 1957, 1958 and 1959 are characterized by very low nitrogen concentrations (mean = 0.9% N, *n* = 3), and relatively depleted values of δ^15^N (mean 4.6‰, *n* = 3) relative to the 20th C average (mean = 5.2‰, *n* = 100). This continues the trend toward lighter δ^15^N in the latter half of the 20th century (1951–2000 = 4.5‰ vs the 1901–1950 mean = 5.8‰).

Values of δ^13^C were also depleted in the dark, calcite-poor sediments deposited through the late 19th C (mean −30‰, *n* = 26) relative to the remainder of the Canadian Zone (mean ~ −28.2‰, *n* = 102). There was a trend toward increasing δ^13^C values until 1948 CE, after which there was a sharp depletion and general decline upcore. Two organic-rich (mean = 16.3% C, 1.4% N, *n* = 17) dark layers with very thin ‘summer’ laminae that are lithologic markers spanning 1950–1956 and 1960–1968 CE across the deep basin of Crawford Lake, are characterized by highly depleted values of δ^13^C (mean −29.3‰, *n* = 17). These dark layers are punctuated by the three prominent calcite laminae assigned to 1957, 1958 and 1959 CE. The much less depleted δ^13^C values (mean = −25.1‰, *n* = 3) in these samples are consistent with inorganic calcite, but low total organic carbon is suggested by the very low total carbon concentrations (mean = 13.6% C, *n* = 3).

## Discussion

The record of bomb radionuclides measured in individual varves from cores collected below the chemocline of Crawford Lake in April 2023 supports the selection of its varved sequence as the GSSP candidate (McCarthy et al., 2023) and refines the varve chronology based on measurements of ^137^Cs at annual resolution across the proposed base of the Anthropocene series/epoch. The plutonium profile was refined using measurements of concentrations of atoms of ^239^Pu and ^240^Pu and resulting ^240^Pu/^239^Pu atom ratios and calculated ^239 + 240^Pu activity in 12 individual varves spanning 1949–1954 and 1961–1966 CE (Figure 6a). The results are consistent with previously analysed samples from Crawford Lake (Figure S1; replotted in Figure 6(b) and (c)) following the revised varve chronology, adding 1 year above the thickest Dust Bowl marker varve, now assigned to 1936 CE. Peak activities of ^239^Pu and ^240^Pu, corrected from concentrations per dry weight using AMS (Table S4), are higher than those measured in previous samples from Crawford Lake freeze cores, but values fall exactly on the trendline of measurements from the 2019 and 2022 cores. The higher values thus reflect analysis of individual varves through the early 1960s that were missing from previous analyses without dilution from combining multiple varves as required for alpha spectrometric determinations.

The plutonium profiles from the varved succession of Crawford Lake record a well-resolved signature of the early nuclear weapons testing period (1945–1954 CE), recording both the upturn of Pu activities following the onset of high-yield thermonuclear weapons testing in 1952, and earlier testing activity. This includes potential tropospheric fallout signals from Nevada and New Mexico (USA) tests, consistent with the location of this unusual meromictic basin in the path of winds from the southwest from the New Mexico and Nevada test sites. The well-oxygenated monimolimnion, in which sediments accumulate without disturbance because bottom water conditions prevent macroscopic benthic organisms from colonising the deep-basin lakebed, may also play a role in preventing mobilization of plutonium in the varved succession, allowing the nuclear moratorium that lasted from November 1958 to September 1961 to be represented by a distinct shoulder, with a sharp decline in ^239 + 240^Pu activity to 0.0104 ± 0.0006 Bq/g in the sample from 1960 CE (Figure 6), not clearly seen in profiles from auxiliary sites in Asia and Europe (AWG, 2024b).

Plutonium measurements from individual varves also fill important gaps, notably leading up to the Limited Test Ban Treaty in late 1963 CE. This is also recorded by a pronounced shoulder spanning several samples in the ^137^Cs profile, preceding the sharp increase at the peak of the Cold War and the subsequent decline (Figure 5). Annual resolution also confirms the very low ^240^Pu/^239^Pu values in seven consecutive samples dating to the latest 1940s though early 1950s, with ratios well below the global fallout value of 0.180 ± 0.014 for mid–latitude sites (Wu et al., 2011), with lowest values in varves assigned to 1951 through 1953 CE using the updated varve chronology. A similar ratio was measured in a grass sample from summer 1952 in the Rothamsted archive, but rising quickly thereafter (Warneke et al., 2002). Low ^240^Pu/^239^Pu ratios indicate a low burn-up of the nuclear fuel which can be attributed to low-yield fission weapons for example, from the Nevada test site (Hicks and Barr, 1984) whereas the thermonuclear explosions at the Pacific Proving Grounds produced ratios well above the global fallout average, that is, ^240^Pu/^239^Pu > 0.21 (Buesseler, 2023). The combination of very low ^240^Pu^/239^Pu (i.e. relatively more ^239^Pu than ^240^Pu fallout) and low but gradually rising plutonium activity provides a useful marker for the base of the proposed Anthropocene series/epoch in the varved sediments of Crawford Lake, consistent with the AWG supermajority decision to employ ^239^Pu, and specifically the ‘initial major rise’ in this anthropogenic isotope, as the primary marker (AWG, 2024a).

**Figure 8.**
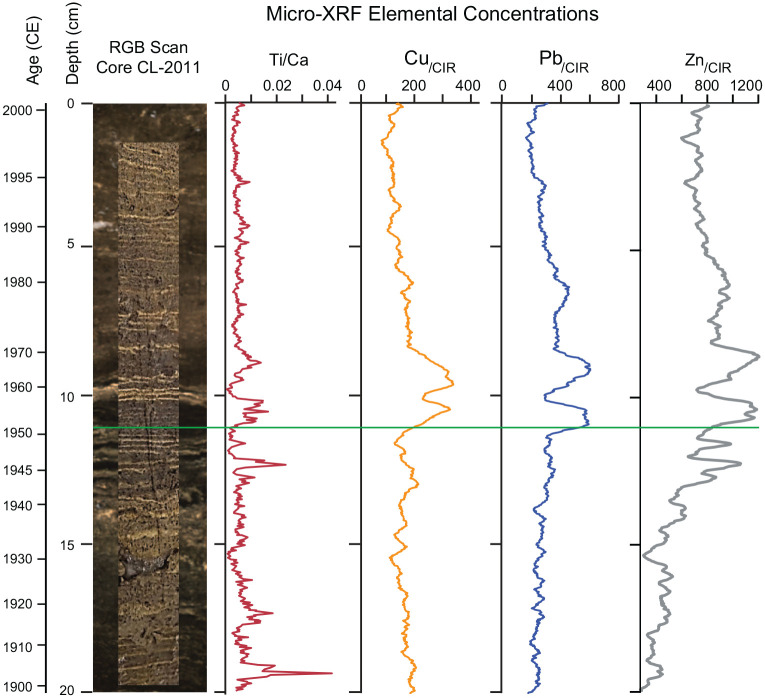
Results of ITRAX micro-XRF analysis of varved sediments from Crawford Lake. Peaks in Ti:Ca are attributed to increased terrigenous sediment influx due to land disturbance, soil erosion and/ or increased precipitation and runoff from the small catchment. Image of core CL-2011 superimposed on the RGB core scan with varve age model from this article. Heavy metals (Cu, Pb, Zn) associated with fly ash are more abundant in sediments deposited between 1952 CE (green line marking the proposed base of the Anthropocene) and 1980 CE and are reduced in calcite-rich (light coloured) laminae as an artifact of the strong contribution of Ca to total elemental abundances.

The shift in the Earth system that led atmospheric chemist Paul Crutzen to insist that we were no longer living in a Holocene world is not attributed to nuclear fallout, but to the overwhelming effects of humans during the Great Acceleration (Steffen et al., 2007, 2015). Key markers of the rapid expansion of the fossil fuel economy and human population growth in the mid-20th century are δ^15^N and SCPs, with sharp acceleration measured at most of the sites studied as potential Anthropocene GSSPs (Waters et al., 2023), including Crawford Lake. Analysis of carbon and nitrogen from individual varves in this study supports the changes in atmospheric composition and associated limnologic and biotic changes, despite the strong influence of water chemistry on varves comprised almost entirely of authigenic organic matter and calcite crystals on measurements of organic proxies (Table 6, Figure 7). Dark sediments with thin ‘summer’ calcite laminae deposited until ~1903 CE are rich in carbon and nitrogen but characterised by highly depleted values of δ^15^N and δ^13^C. The scarcity of calcite in these sediments is attributed to lower pH due to effluent from the lumber mill (rich in plant matter and humic acid) resulting from intense logging in the Crawford Lake catchment and operation of a lumber mill at its outlet between ~1882 and 1900 CE (McCarthy et al., 2023). The effects of land use on organic proxies remained clear even after closure of the lumber mill, when the lake became a private recreational area for the extended Crawford family. A sharp negative anomaly in %C and %N, accompanied by highly depleted values of δ^13^C, for instance, correlates with a sharp increase in ratios of titanium: calcium (Figure 8) and deposition of an anomalous ‘non-varve’ unit. The decrease in organic proxies and increase in Ti:Ca is indicative of increased terrigenous sediment influx into this deep karstic basin (Llew-Williams et al., 2024), perhaps associated with decommissioning of the mill. Gushulak et al. (2022) reported an increase in chrysophyte diversity succeeding the nearly monospecific *Mallomonas pseudocoronata* assemblage that characterised the ‘lumbering era’ in the Crawford Lake catchment.

Later activities in the catchment are also recorded by organic proxies, notably in 1969–1971 CE, when another non-varve unit was deposited. This coincides with the establishment of the Conservation Area and associated changes in the water column are recorded by a sharp change in both diatom and chrysotphyte assemblages (Marshall et al. 2023) and algal palynomorphs (McCarthy et al., 2023).

**Figure 9.**
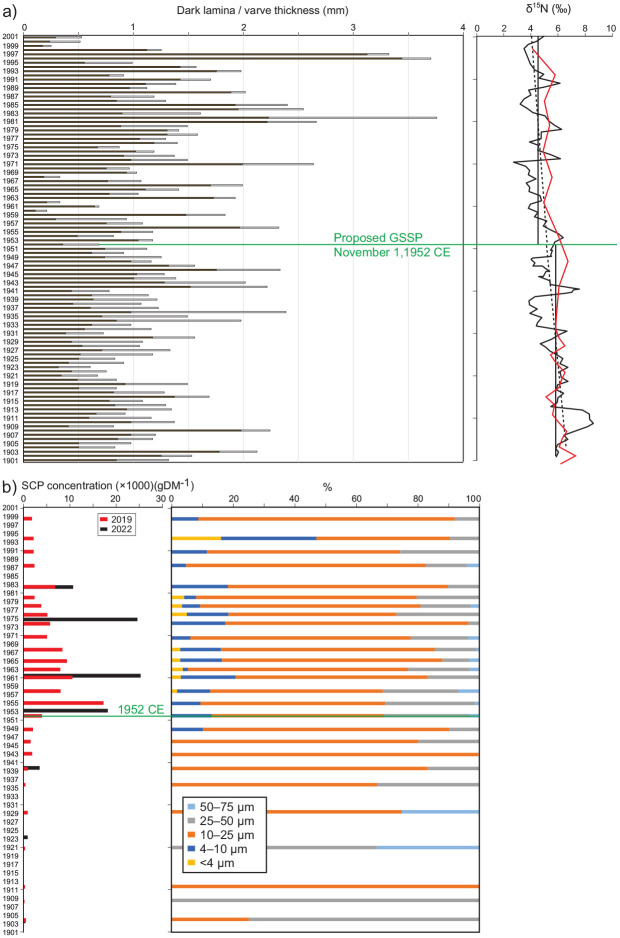
(a) Measurements of δ^15^N on individual varves from core CRA23-2FT-A1 show a gradual decline (dashed line), particularly beginning in the second decade of the 20th C, illustrating the global effects of mass-produced automobiles and nitrogen fixing using the Haber-Bosch method, and the regional effects of steel mills. The red line shows a similar trend measured in samples combining multiple varves, published in McCarthy et al. (2023) but since corrected for a spreadsheet error at U Leicester. The lower δ15N mean through the proposed Anthropocene (4.46‰) to the post-1952 CE mean (5.15‰, *n* = 100). (b) High concentrations of fly ash in freeze cores CRA19-2FT-B2 (red) and CRA22-1FR-A (black) reflect the rapid increase in high temperature combustion of fossil fuels during the Great Acceleration, particularly at steel mills in nearby Hamilton where half of Canada’s steel was produced during the post-WWII boom, although higher relative abundances of smaller particles since record more distal influence in the latter half of the 20th C.

Extra-local signatures are captured by long-term trends, such as that toward increasingly depleted values of δ^15^N though the 20th century (Figures 7 and 9a). These signatures are attributed to increased emission of greenhouse gases, the primary factor responsible for the departure of modern conditions from Holocene norms (Summerhayes et al., 2024). The sharp decline in values of δ^15^N around 1912 CE, for instance, may be attributed to invention of the energy-intensive Haber-Bosch process for fixing nitrogen from the atmosphere (Darmawan et al., 2020) in 1910 and the establishment of the Steel Company of Canada (STELCO) and Dominion Foundries and Steel (DOFASCO) in Hamilton in 1910 and 1912, as well as the mass production of automobiles beginning in 1908 CE.

Mean values of δ^15^N 1.3‰ are lighter in the latter half of the 20th century than during 1901–1952 CE, with increasingly depleted values upcore superimposed on lithological variations. The proposed Anthropocene series/epoch is defined by a trend beginning in 1953 CE to the most depleted values of the 20th C (2.70‰) measured in the thick varve deposited in 1971 CE (Figure 9a). This correlates with sharply higher concentrations of SCPs in the varved sediments of Crawford Lake beginning in 1952 CE (Figure 9b) and increased abundances of zinc, lead and copper (Figure 8) from fly ash particles formed when coal and oil are combusted at high temperatures (Davison et al., 1974). Extra-regional influx of fly ash can also be inferred from the higher relative abundance of smaller SCPs in the latter half of the 20th C, even after the absolute abundance/ concentration of SCPs declined in response to more stringent air quality standards beginning in the 1970s (Figure 9b).

## Conclusions

Analysis of Anthropocene markers from individual varves from the deep basin of the meromictic Crawford Lake support the proposal for a GSSP at 17.5 cm in core CRA23-BC-1F-B (AWG, 2024b). The contact between the thin, light-coloured lamina deposited in the summer of 1952 CE and the overlying dark-coloured lamina primarily recording organic matter reaching the lakebed during fall turnover correlates with detonation of the first H-bomb, ‘Ivy Mike’ at Enewetak Atoll in the Marshall Islands. The precise moment of detonation, 7:15AM local time on November 1, 1952, nominally represents the onset of the proposed Crawfordian Age and Anthropocene Epoch, much like the moment of asteroid impact nominally marks the onset of the Cenozoic Era. The initial major rise in activity of ^239 + 240^Pu occurs in the sample assigned a varve age of 1952 CE, an increase of 0.0031 Bq/g relative to 1951, and the lowest values of the ^240^Pu/^239^Pu atom ratio were measured in this, 0.069 ± 0.007 in 1952 CE. This provides the chronostratigraphic basis for identifying the base of the proposed Anthropocene epoch.

## Supplemental Material

sj-pdf-1-anr-10.1177_20530196251315454 – Supplemental material for High-resolution analysis of the varved succession at Crawford lake across the base of the proposed Crawfordian stage and Anthropocene series Supplemental material, sj-pdf-1-anr-10.1177_20530196251315454 for High-resolution analysis of the varved succession at Crawford lake across the base of the proposed Crawfordian stage and Anthropocene series by Francine MG McCarthy, R Timothy Patterson, Carling Walsh, Krysten M Lafond, Brian F Cumming, Andy B Cundy, Karin Hain, Pawel Gaca, Peter Steier, Arnoud Boom, Paul B Hamilton, Michael FJ Pisaric, Martin J Head, Joseph I Boyce, Neil L Rose and Simon D Turner in The Anthropocene Review
