# Supplementary material for: High-resolution analysis of the varved succession at Crawford lake across the base of the proposed Crawfordian stage and Anthropocene series
Source: Anthropocene Rev. 2025 Mar 31;12(2):243–72. doi: 10.1177/20530196251315454 (PMC12317751; doi:10.1177/20530196251315454)
Supplement: sj-pdf-1-anr-10.1177_20530196251315454 – Supplemental material for High-resolution analysis of the varved succession at Crawford lake across the base of the proposed Crawfordian stage and Anthropocene series [file sj-pdf-1-anr-10.1177_20530196251315454.pdf]

Supplementary Information: High-resolution analysis of the varved succession of Crawford Lake across the proposed base of the Anthropocene

This article refines the case made by McCarthy et al. (2023) for the varved succession from Crawford Lake to define the Anthropocene. Its selection among 11 other potential candidate GSSPs and reference sections was announced at a press conference in Lille, France in June 2023, a little less than a year after publication of proposals in a special volume of *The Anthropocene Review* (Waters et al., 2023). When the sediment succession from the deep basin of this meromictic lake in southern Canada was selected by a supermajority of Anthropocene Working Group voting members, individual varves had been subsampled at annual resolution for several key ‘Anthropocene proxies.’ These were previously only available at lower resolution from the varved sediments (**Figure S1**), but their sudden rapid change along with the prominent visibility of the annual lamination made a particularly strong argument for the ‘golden spike’ together with its protected yet accessible location where there is clear evidence of anthropogenic impact by Indigenous agricultural settlement well before the tipping point in the Earth system postulated by Paul Crutzen.

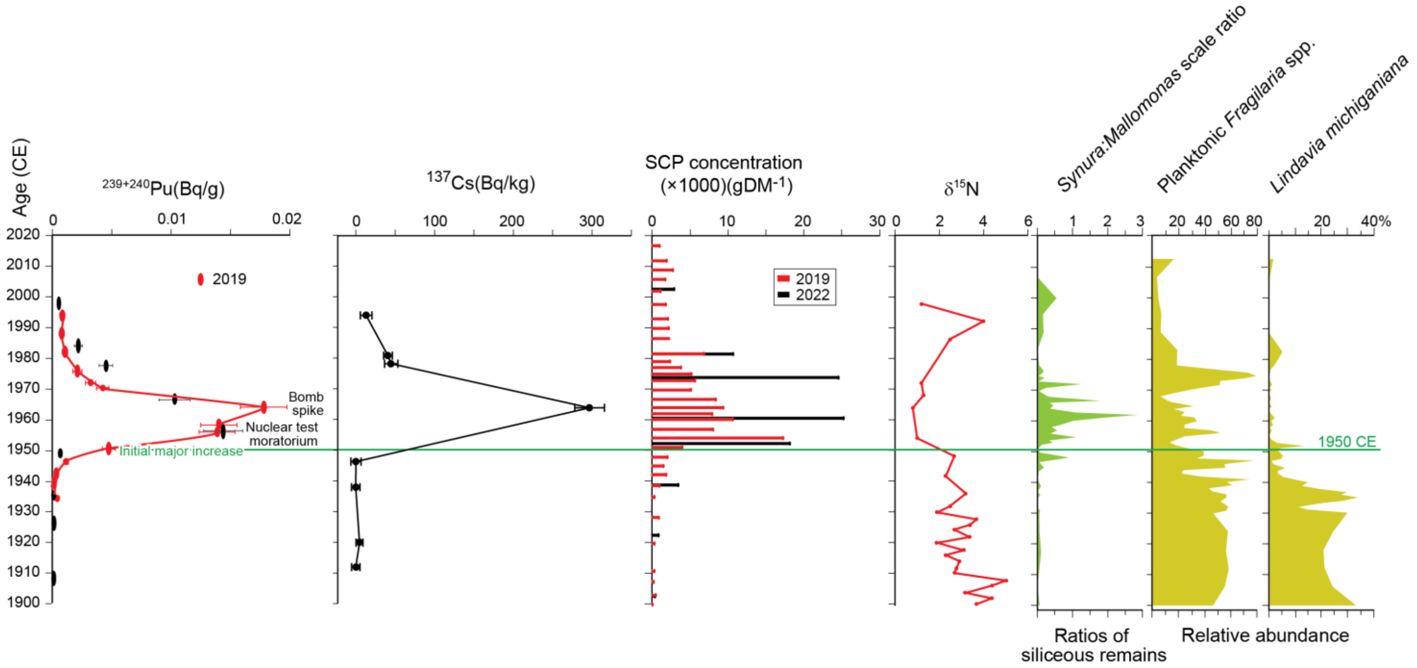

Figure S1. The impact of nuclear arms testing through the Cold War ($^{239+240}$Pu and ^{137}Cs) and rapidly increased fossil fuel combustion (SCPs/ fly ash and δ^{15}N) during the Great Acceleration are clear in varve-age dated samples from Crawford Lake between 1950 and 1975 CE (data from freeze cores collected in 2019 in red and 2022 in black, ellipses representing varves combined for plutonium analysis). The effects of acid rain on water chemistry are evident in the shift in microfossil remains of siliceous algae: the increase in deep dwelling chrysophytes (green) and decline in planktonic diatoms (yellow) reflect the decline in calcite precipitation in the epilimnion of Crawford Lake, allowing deeper light penetration. Modified from McCarthy et al. (2023), showing the previously suggested base for the Anthropocene series/ epoch in the varve deposited in 1950CE (at 15.6 cm in core CRA22-1FR-3, illustrated in **Figure 2**).

Two full days of freeze coring took place in exceptionally mild (low 20s °C) and low wind conditions. The GPS location of each core face recovered on April 12–13, 2023 and the length of each measured in the field can be found in **Table S1**. Photographs of the core faces sampled for analysis (including the core face proposed as the Anthropocene GSSP, the 83 cm-long one-face core CRA23-BC-1F-B) are shown in **Figure S2**, illustrating the ease with which individual varves can easily be traced across all cores and in 3-dimensions, particularly by the trained eye.

Table S1. Freeze cores recovering proposed Anthropocene sediments collected from the deep basin of the meromictic Crawford Lake in April 2023. Note: 2FT = two face corer, 1FT – one-face corer. **Bold** = core faces subsampled for analysis across the proposed base of the Anthropocene, shading = proposed GSSP core, archived at the National Biodiversity Cryobank of Canada, Canadian Museum of Nature, Ottawa Ontario.

**Date of collection**	**Core name**	**Location (UTM zone 17T) Easting, Northing**	**Core length (cm)**
**2023/04/12, 8AM**	**CRA23-2FT-A-F1**	**585032, 4813411**	**127**
**2023/04/12, 8AM**	**CRA23-2FT-A-F2**	**585032, 4813411**	**117.5**
2023/04/12, 10AM	CRA23-2FT-B-F1	585030, 4813409	86.5
2023/04/12, 10AM	CRA23-2FT-B-F2	585030, 4813409	86.5
**2023/04/13, 8:30AM**	**CRA23-BC-1F-A**	**585028, 4813407**	**89**
**2023/04/13, 11AM**	**CRA23-BC-1F-B**	**585029, 4813406**	**83**
2023/04/13, 2:30PM	CRA23-BC-1F-C	585027, 4813409	111.5
2023/04/13, 4:00PM	CRA23-BC-1F-D	585029, 4813406	87

CRA23-BC-1F-A

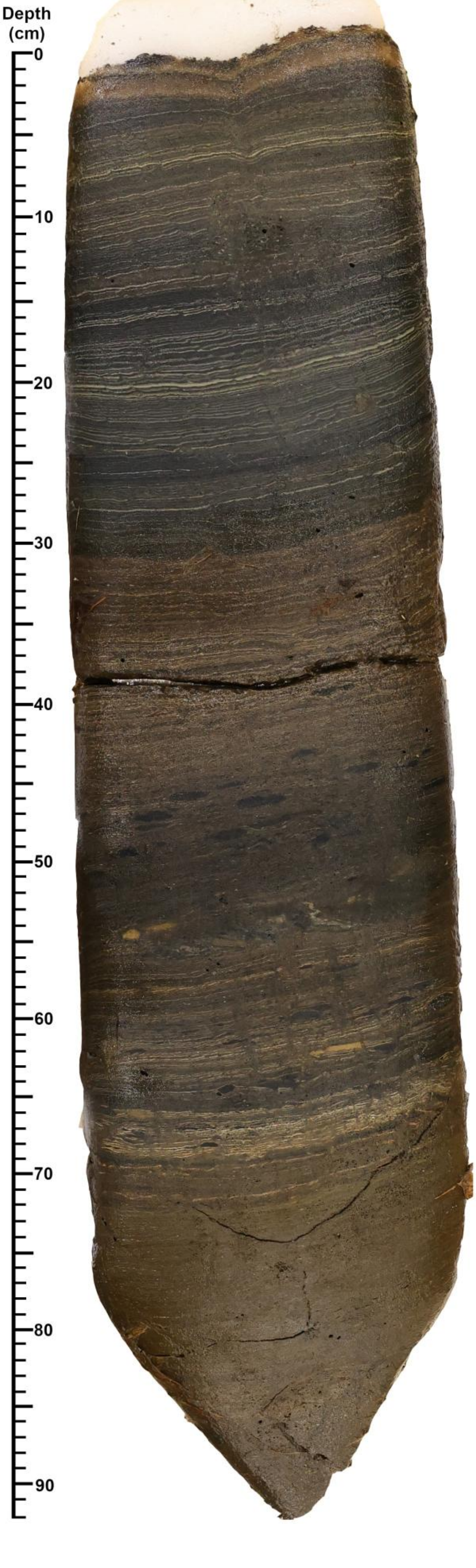

CRA23-BC-1F-B

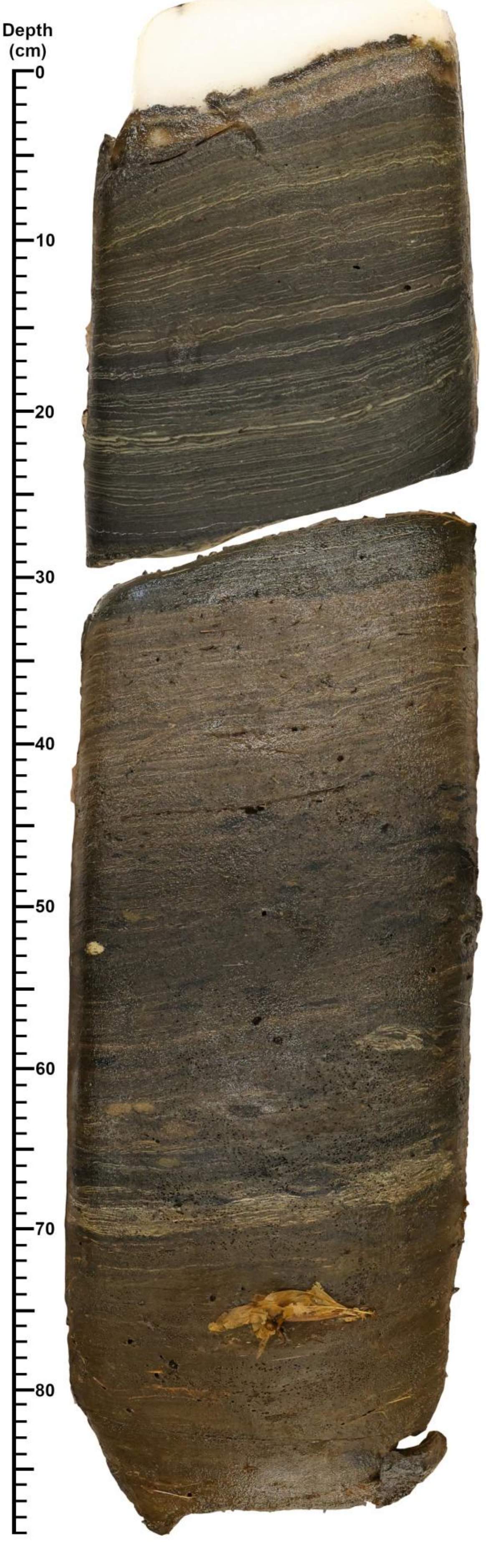

CRA23-2FT-A-F1

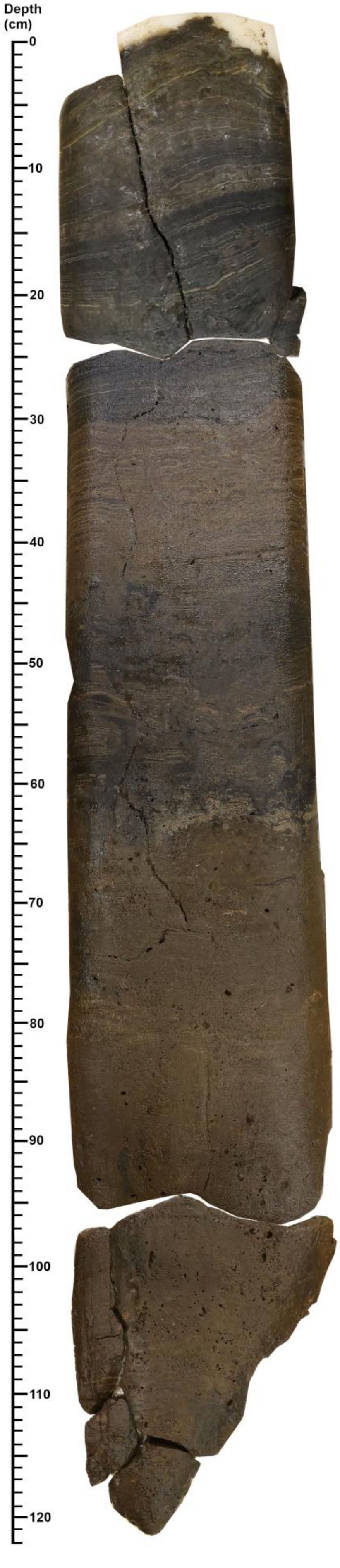

CRA23-2FT-A-F2

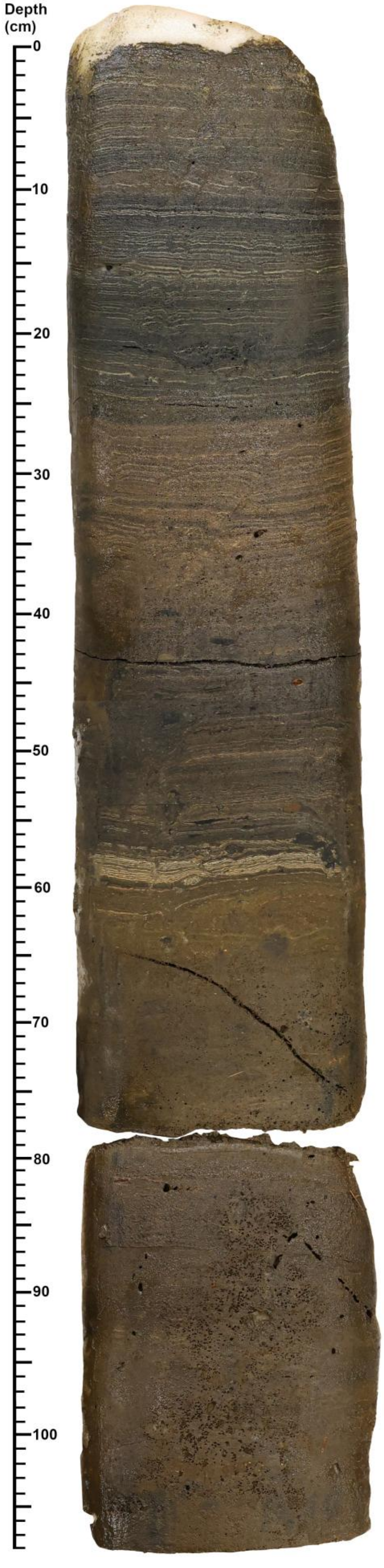

Figure S2. Two of the one-face cores CRA23-BC-1F-A and -B and one face of the longer, two-faced core CRA23-2FT-A were subsampled at annual resolution, each varve meticulously sampled along three-dimensional faces that not only slope slightly but also bend along the thickness of the face as the sampler slowly subsided through the sediment. The photographs illustrate the distinctive varve pattern that allows confident correlation across cores through the Canadian Zone (since 1867 CE, just below the colour change around 30 cm that is attributed to intensive logging and lumber milling dated to 1879 CE using the varve chronology described in this paper. Seasonal (summer) precipitation of calcite began when primary production caused the pH of surface waters to rise in response to cultural eutrophication; this is recorded by pollen of cultigens and spores of their pathogens in sediments beginning in the late 13th century (Ekdahl et al., 2004, 2007; McCarthy et al., 2023; Llew-Williams et al., 2024) and confirmed by archaeological excavations (Byrne and Finlayson, 1975; Finlayson, 1998).

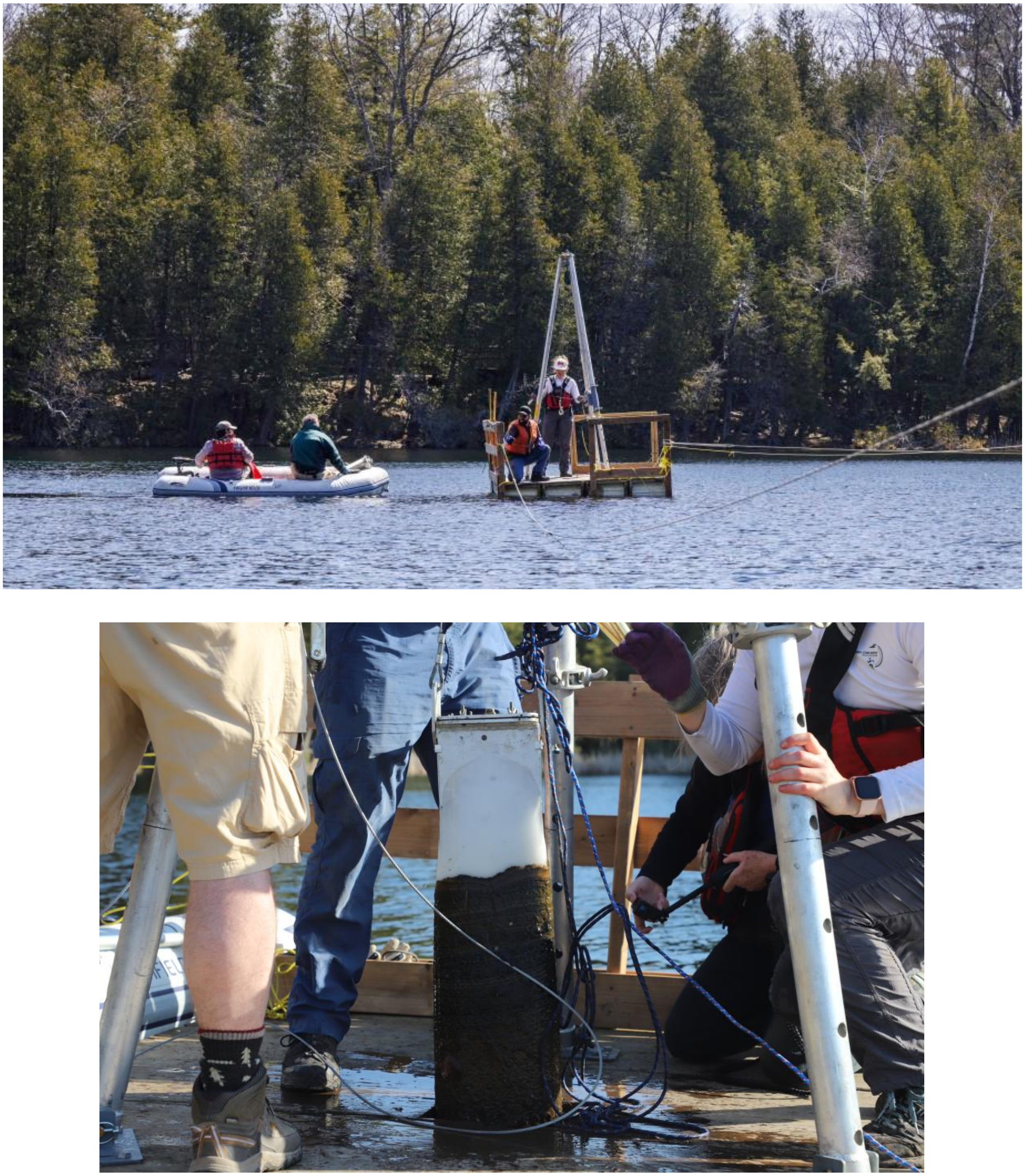

Figure S3. Field work, April 12–13, 2023. Because safe ice did not form during the winter of 2023, the *Grampie Roy* platform (Patterson Laboratory, Carleton University) was fixed above the deep basin of Crawford Lake using 5 lines (some of which are visible in the drone photo above). Several freeze corer models were deployed through the moon pool to collect sediments in April 2023, one of which is the single face sampler from Queen's University shown in the lower photo above, that was sampled for plutonium analysis at annual resolution in this study. Photos below illustrate transportation of the freeze core ashore (a) and release of the core face from the sampler (b-d) before packing on more dry ice before being transported to the Patterson Laboratory at Carleton University, Ottawa.

a
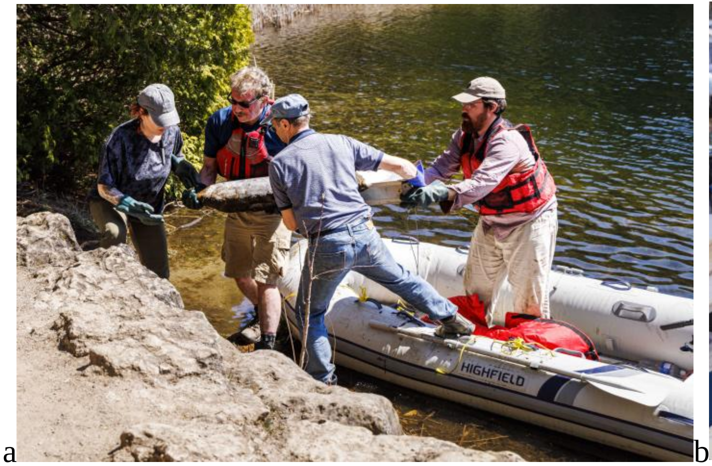

b
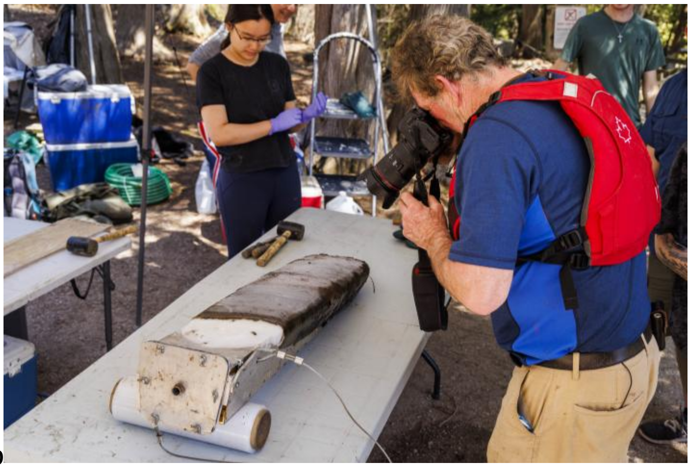

c
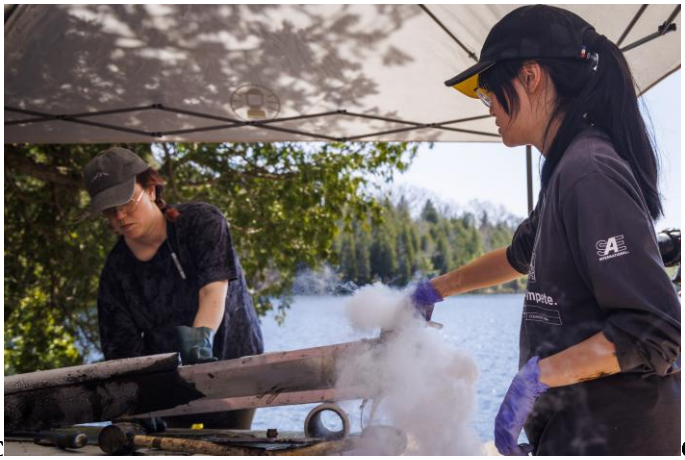

d
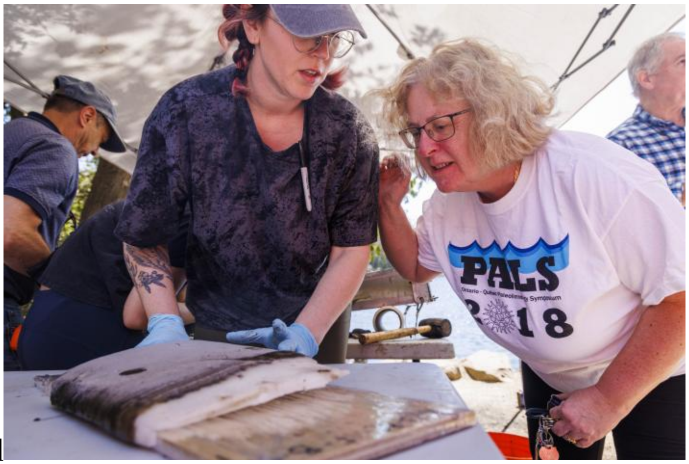

Figure S4 (below). Krysten Lafond meticulously subsampling individual varves in frozen core faces in the cold storage area of the Patterson Laboratory at Carleton University in May 2023. Varve boundaries are carefully followed in three dimensions and sampled with a scalpel from the base of the light lamina to the top of the overlying dark lamina, since most sediment accumulates during the growing season and during fall turnover, best identifying the calendar year of accumulation. Sample vials were labelled following the varve age chronology of Lafond et al. (2023) and shipped to labs where analysis of cores from 2011, 2019, and 2022 were carried out (McCarthy et al., 2023).

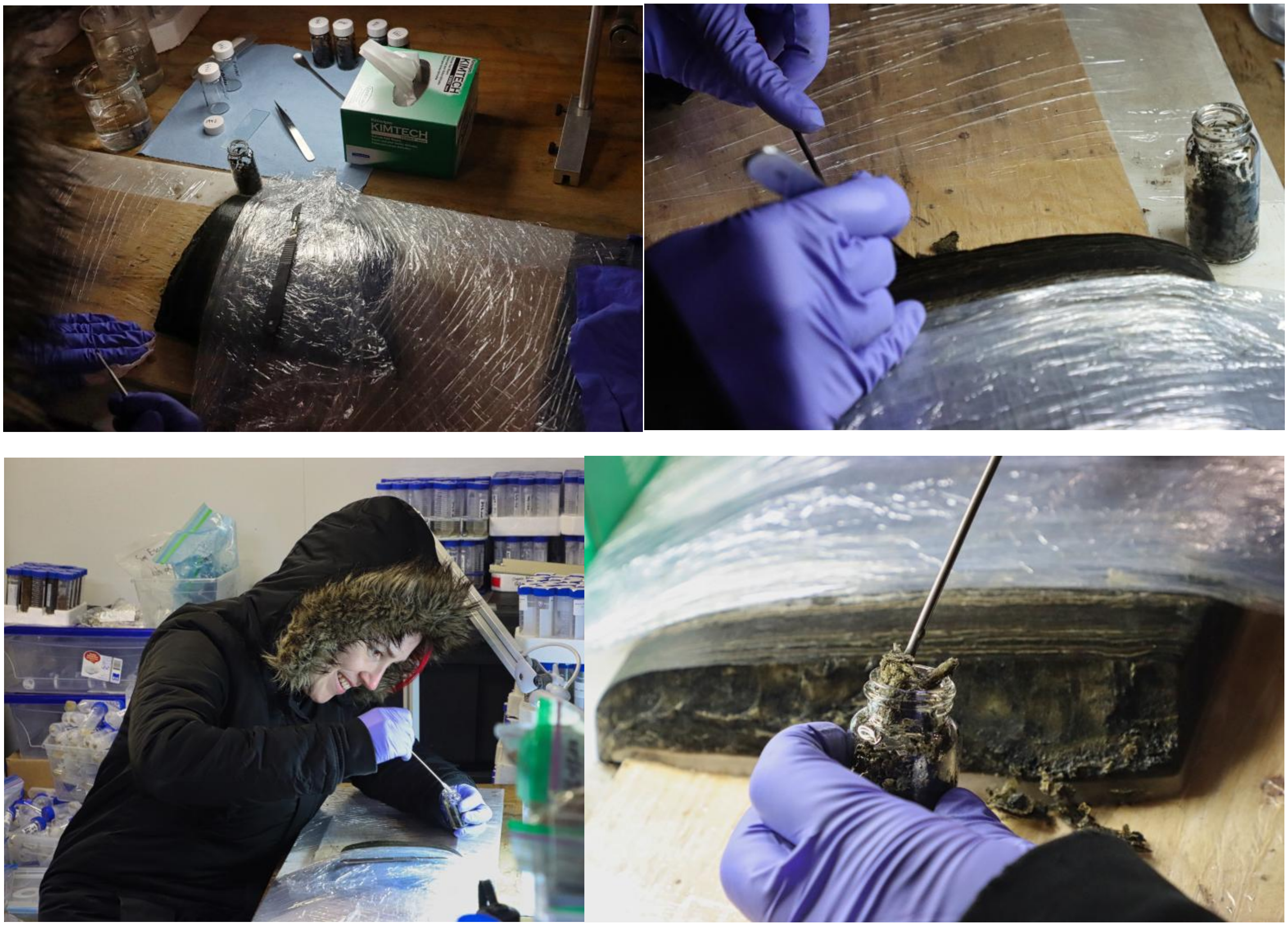

Laboratory reports are provided as supplementary material – from Queen's University below and as Excel files from the University of Leicester and University of Vienna, whereas samples prepared for plutonium analysis at the University of Southampton were sent for AMS analysis. Results of ^{137}Cs analysis (**Table S2**) suggested that the varve ages were slightly older than they should be, leading to re-examination of varves in all freeze cores collected between 2019 and 2023 and re-assessment of the basis for assigning the thick marker varve (originally assigned to 1935 and now considered to be 1936 CE) from which calendar ages were assigned up- and downcore (Lafond et al., 2023).

Table S2. Gamma ray analysis, core CRA23-2FT-A B. Cumming, analyst, PEARL, Queen's University. Light shading highlights ^{137}Cs activity 50–150 Bq/kg, and dark shading over 150 Bq/kg.

Chn file	Year	Mdpt	Dry Sediment (g)	tube height Cm	137-Cs [Bq/kg]	137-Cs (error) [Bq/kg]
CRA-23 1970 (92x) (160000 sec)	1970	1970.5	0.27	0.75	16.17	5.53
CRA-23 1969 (92x) (160000 sec)	1969	1969.5	0.11	0.68	27.69	13.02
CRA-23 1968 (92x) (160000 sec)	1968	1968.5	0.16	0.57	19.35	8.95
CRA-23 1967 (92x) (160000 sec)	1967	1967.5	0.18	0.69	37.22	8.46
CRA-23 1966 (92x) (160000 sec)	1966	1966.5	0.10	0.59	70.56	15.28
CRA-23 1965 (92x) (160000 sec)	1965	1965.5	0.05	0.70	100.5	30.01
CRA-23 1964 (92x) (160000 sec)	1964	1964.5	0.08	0.53	225.25	24.16
CRA-23 1963 (92x) (160000 sec)	1963	1963.5	0.14	0.63	189.62	16.81
CRA-23 1962 (92x) (160000 sec)	1962	1962.5	0.04	0.54	265.22	42.25
CRA-23 1961 (92x) (160000 sec)	1961	1961.5	0.21	0.66	103.21	10.18
CRA-23 1960 (92x) (160000 sec)	1960	1960.5	0.04	0.59	2.54	34.26
CRA-23 1959 (92x) (160000 sec)	1959	1959.5	0.03	0.33	43.99	45.76
CRA-23 1958 (92x) (160000 sec)	1958	1958.5	0.17	0.69	70.17	10.12
CRA-23 1957 (92x) (160000 sec)	1957	1957.5	0.07	0.53	23.2	19.63
CRA-23 1956 (92x) (160000 sec)	1956	1956.5	0.45	1.05	59.65	5.32
CRA-23 1955 (92x) (160000 sec)	1955	1955.5	0.13	0.72	44.12	11.61
CRA-23 1953-54 (92x) (160000 s)	1953.5	1954	0.04	0.49	0	32.99
CRA-23 1952 (92x) (160000 sec)	1952	1952.5	0.05	0.48	0	26.4
CRA-23 1950 (92x) (160000 sec)	1950	1950.5	0.08	0.59	0	16.5
CRA-23 1949 (92x) (160000 sec)	1949	1949.5	0.06	0.64	4.23	22.84
CRA-23 1948 (92x) (160000 sec)	1948	1948.5	0.24	0.75	0	5.5
CRA-23 1947 (92x) (160000 sec)	1947	1947.5	0.14	0.62	0.73	9.79
CRA-23 1946 (92x) (160000 sec)	1946	1946.5	0.13	0.51	0	10.15
CRA-23 1945 (92x) (160000 sec)	1945	1945.5	0.09	0.50	0	14.66

Although the conditions controlling the accumulation of endogenic calcite and dominantly authigenic organic matter below the chemocline of Crawford Lake are complex, water column studies, including sediment trap analysis, quantified hydrological conditions needed for calcite to precipitate in the epilimnion (Llew Williams et al. 2024). Findings support the observation that climate has a strong control, based on wavelet analysis of the thickness of the light and dark-coloured laminae through the Canadian Zone (Lafond et al. 2023). This is reflected in the thick, prominent laminae deposited through the abnormally dry and warm 1930s (total annual precipitation 735.1 mm and mean annual temperature/ MAT 7.9°C), following and preceding cooler, wetter intervals (annual precipitation 809.1 mm and MAT 7.1°C 1915–1929 CE and 832.9 mm and MAT 7.3°C 1940–1951 CE) characterised by thinner calcareous laminae (**Figure 9**; varve thickness was measured from ultra high resolution images of core CRA19-FT-B2 – see **Table S1** in McCarthy et al., 2023).

Llew-Williams et al. (2024) highlighted the importance of primary production in the water column through the summer season (June, July, August, September: JJAS) in addition to rainfall as key factors affecting pH, one of the key variables controlling the precipitation of calcite, together with water temperature (**Figure S5**). As a result, greater emphasis was placed on summer meteorological variables in assessing the probable age of the thickest white lamina of the Dust Bowl interval, and antecedent conditions were considered (**Table S3**). The slightly revised age model based on assigning this lamina to 1936 rather than 1935 CE is consistent with the radionuclide records now available from individual varves.

Table S3. Meteorological data from Toronto Station 6158350 (longitude -79.4, latitude 43.67), showing hydrologic year averages for annual temperature and precipitation and temperature and rainfall for the months when the minimum temperature required for crystallization of calcite is achieved, producing the light-colored 'summer lamina'. Gray shading highlights the consecutive relatively warm, dry years leading up to **1936 CE** (**bold**), to which the thickest white lamina is attributed in the revised age model, as well as 1948 and 1949, when the last prominent white lamina were deposited prior to the distinctive 'triplet, assigned to 1957, -58 and -59. The proposed GSSP year, 1952, is also highlighted in bold.

Year CE	Mean Annual Temperature (°C)	Mean Temperature JJAS (°C)	Mean Max Temperature JJAS (°C)	Total Rain JJAS (mm)	Total Annual Precipitation (mm)
1867	7.1	18.5	23.8	166.3	853.2
1868	6.9	18.7	23.6	218.3	823.9
1869	6.3	16.9	21.4	441.0	1027.9
1870	7.5	19.0	24.0	514.5	1187.7
1871	7.4	17.2	22.5	221.8	858.6
1872	6.4	18.7	24.1	264.5	611.4
1873	5.6	17.6	22.9	192.6	818.0
1874	6.5	18.4	23.9	179.7	692.8
1875	5.1	16.9	22.5	212.1	671.2
1876	6.9	18.6	23.7	186.9	833.3
1877	6.7	18.9	24.1	183.6	626.7
1878	9.0	19.1	23.8	567.6	1170.4
1879	6.4	17.3	22.4	311.3	781.7
1880	8.5	18.4	23.3	368.4	966.8
1881	6.7	19.6	24.7	174.3	629.4
1882	7.8	18.0	23.0	211.4	722.1
1883	5.6	16.4	21.5	375.3	899.0
1884	6.4	17.8	23.5	245.4	672.7
1885	5.0	16.7	22.0	352.5	815.7
1886	6.7	17.2	22.6	276.1	910.7
1887	6.6	18.3	23.7	165.7	641.4
1888	5.8	17.5	23.1	281.3	701.5
1889	7.3	17.7	22.5	237.3	700.6
1890	7.7	17.7	22.8	353.0	949.1
1891	7.3	18.0	23.3	299.2	863.4
1892	7.4	18.6	23.7	392.5	837.5
1893	6.4	18.3	23.8	282.6	847.7
1894	8.0	18.9	24.3	217.4	908.7
1895	7.2	18.5	24.1	222.4	602.3
1896	7.4	18.2	23.6	242.3	830.7
1897	7.7	18.5	23.9	276.3	746.6

1898	8.9	20.0	25.4	165.6	736.2
1899	7.5	18.5	24.6	181.1	820.0
1900	8.3	19.9	25.7	237.8	785.4
1901	8.5	19.7	25.2	314.8	849.3
1902	7.5	17.7	23.0	347.7	768.3
1903	8.2	17.8	23.0	299.2	798.0
1904	6.2	17.5	23.0	418.2	958.9
1905	6.7	18.8	24.1	352.1	705.9
1906	8.6	20.1	25.5	324.8	751.2
1907	6.9	18.4	23.9	255.2	766.9
1908	7.9	19.5	25.6	254.6	898.0
1909	8.2	18.7	24.3	202.8	774.9
1910	8.3	18.9	24.6	305.3	873.5
1911	8.3	19.4	25.0	232.2	648.2
1912	7.0	18.2	23.4	279.2	922.0
1913	8.8	18.8	24.9	185.4	713.3
1914	8.4	19.0	24.5	252.4	722.9
1915	8.2	18.4	23.7	463.1	872.3
1916	8.3	19.7	25.5	201.1	790.4
1917	7.1	18.1	23.7	348.6	856.4
1918	6.7	18.2	24.0	367.2	899.9
1919	9.7	20.8	26.7	254.1	806.4
1920	7.3	19.2	24.9	243.0	638.6
1921	10.5	21.5	27.4	207.2	750.5
1922	8.8	19.6	25.4	313.6	846.8
1923	7.4	18.7	24.2	310.5	756.3
1924	7.6	17.6	23.0	357.8	965.1
1925	8.1	19.1	24.3	302.9	682.8
1926	6.6	17.7	22.7	433.7	939.0
1927	7.6	18.2	23.7	278.8	751.8
1928	8.1	18.4	23.5	443.7	931.1
1929	8.4	18.4	24.0	218.6	950.0
1930	8.3	19.7	25.3	235.3	766.2
1931	9.1	20.4	25.6	242.8	600.9
1932	9.5	19.2	24.2	299.6	921.2
1933	9.4	20.1	25.7	181.2	610.2
1934	7.2	19.5	24.7	243.7	658.1
1935	8.1	19.3	24.2	270.9	683.2
**1936**	**8.0**	**19.7**	**25.3**	**211.6**	**704.0**
1937	8.8	19.8	24.9	275.2	857.2
1938	8.7	19.7	24.9	257.9	691.2
1939	8.8	20.0	24.9	262.2	736.7
1940	7.6	19.0	23.8	297.3	724.5
1941	8.8	20.1	25.3	232.9	729.9
1942	9.3	19.4	24.2	301.7	910.8
1943	8.0	19.7	24.7	249.0	941.4
1944	8.7	20.1	25.1	279.2	751.0
1945	8.3	19.0	23.7	386.3	1015.4
1946	8.6	19.0	24.2	233.4	727.8
1947	8.8	20.0	24.8	363.9	915.4
1948	8.8	20.4	25.6	215.7	652.6
1949	10.0	20.9	26.1	204.5	662.2
1950	8.6	18.8	23.7	284.5	802.0
1951	9.3	19.5	24.4	278.9	881.7

**1952**	**9.3**	**20.5**	**25.6**	**192.1**	**764.5**
1953	9.9	20.3	25.4	262.9	731.4
1954	9.4	19.5	24.2	239.7	691.4
1955	10.1	21.3	26.1	264.5	844.3
1956	8.0	18.6	23.1	324.1	960.3
1957	9.3	19.7	24.4	312.9	777.0
1958	9.0	19.0	23.8	326.0	707.8
1959	9.1	21.7	26.6	185.7	684.8
1960	8.7	19.8	24.6	212.4	926.2
1961	8.9	20.4	25.2	309.5	809.4
1962	9.1	19.3	23.9	353.2	682.4
1963	8.2	19.2	24.4	203.9	679.2
1964	9.3	19.2	24.0	318.3	760.6
1965	8.4	18.7	23.4	233.4	750.7
1966	8.9	20.1	25.0	207.1	732.7
1967	8.5	19.5	24.1	351.0	907.0
1968	8.6	19.8	24.6	360.6	903.6
1969	9.1	20.2	24.9	192.1	700.7
1970	8.7	20.5	24.9	295.0	753.9
1971	8.9	20.3	24.6	233.8	688.2
1972	9.1	19.6	23.5	330.1	854.2
1973	9.6	21.2	25.1	217.2	858.9
1974	9.0	19.6	23.6	275.5	959.0
1975	9.6	20.2	24.1	303.8	792.5
1976	9.5	19.7	23.6	284.0	877.8
1977	8.5	19.7	23.6	510.5	891.2
1978	8.2	19.4	23.7	269.0	872.3
1979	8.3	19.2	23.4	211.8	758.1
1980	8.9	19.4	23.4	267.0	888.3
1981	8.5	19.5	23.2	401.6	822.5
1982	8.0	18.5	22.4	403.6	947.2
1983	10.1	21.0	25.4	196.3	796.1
1984	8.4	19.7	23.7	235.8	892.0
1985	9.3	19.3	23.3	271.6	772.4
1986	9.0	19.0	22.8	578.3	1127.7
1987	10.2	20.7	24.8	297.4	781.0
1988	9.6	20.9	25.5	230.2	656.2
1989	9.2	20.3	24.1	270.4	678.7
1990	9.2	20.0	24.0	291.6	835.8
1991	10.5	21.0	25.2	288.6	949.3
1992	8.6	18.0	21.9	359.8	832.0
1993	8.9	19.7	23.8	327.2	899.6
1994	8.4	20.1	24.2	204.6	718.6
1995	10.0	20.7	24.7	268.0	780.2
1996	8.4	20.1	24.0	499.2	1171.0
1997	8.9	19.8	24.0	231.8	786.4
1998	11.0	21.0	25.4	219.4	725.8
1999	10.8	21.5	25.7	278.2	676.4
2000	10.0	19.5	23.5	304.6	798.0
2001	9.8	20.8	25.2	225.0	722.2
2002	11.2	22.0	26.7	215.4	799.4
2003	4.7	18.7	23.2	84.8	595.1

In association with re-examination of the varves across all freeze core faces, the meteorological observations supported adjustment of the varve chronology that became evident when measurements of both $^{239+240}Pu$ and ^{137}Cs were available, placing the highest bomb fallout in 1963 rather than in 1962 CE. As a result, one calendar year was added to each data point above the (now 1936 CE) marker varve and adjusting accordingly to compare with previous analyses from Crawford Lake; plutonium analyses from cores collected in 2019 and 2022 integrated with annual measurements performed in 2023 in **Table S4**.

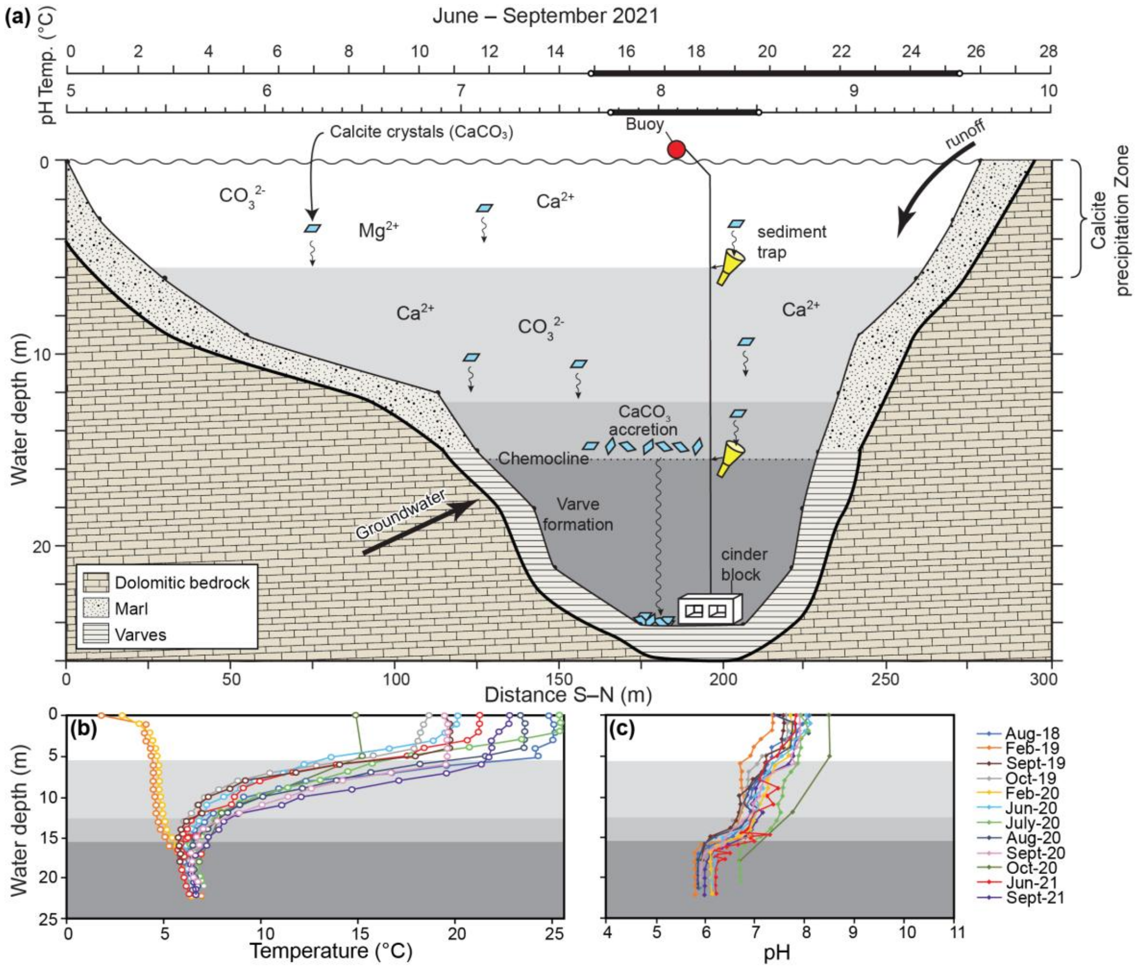

Figure S5. Temperatures warmer than 15°C and pH above 7.8 were only measured in the upper 5.5 m of the water column between June and September, when high primary production sequestered carbon in the epilimnion of Crawford Lake. The volume of calcite precipitated in the epilimnion is critical to the formation of light-coloured laminae since crystals must survive sinking through the slightly acidic monimolimnion, explaining the breakdown of varve chronology between intervals of cultural eutrophication. Modified from Llew-Williams et al. (2024).

Table S4. Results of all plutonium analysis on varved sediments from Crawford Lake, with original varve ages (sample IDs) following the model of Lafond et al. (2023) but revised varve age (+1 year added to conform with ^{137}Cs measurements at annual resolution) used to plot and interpret all data in this article, in the final column (shaded gray). **Bold identifies measurements from individual varves in 2023,** *and italics identify measurements from the freeze core collected in 2022.* Shading highlights the initial major rise in $^{239+240}$Pu activity and lowest $^{240/239}$Pu ratio in the varve deposited in 1952 CE and the peak in $^{239+240}$Pu activity in 1963 CE followed by higher $^{240/239}$Pu values upcore.

GAU ID	Original ID	Pu-239+240 [Bq/g-dry]	+/-	Pu-240/ Pu-239 [Bq/g-dry]	+/-	Final varve age (yCE)
GAU4419/1	*CRA22-1FT-3 1906-1911*	*0.0001*	-	-	-	*1907-1912*
GAU4419/2	*CRA22-1FT-3 1924-1929*	*0.0001*	-	-	-	*1925-1930*
GAU4200/6-1	CRA19-2FT-D1 1934-35	0.0004	-	-	-	1935-1936
GAU4419/3	*CRA22-1FT-3 1934-1937*	*0.0001*	-	-	-	*1935-1938*
GAU4200/6-2	CRA19-2FT-D1 1938-39	0.0001	-	-	-	1939-1940
GAU4200/6-3	CRA19-2FT-D1 1940-41	0.0002	-	-	-	1941-1942
GAU4200/6-4	CRA19-2FT-D1 1942-45	0.0003	-	-	-	1943-1946
GAU4200/6-5	CRA19-2FT-D1 1946-47	0.0012	0.0002	0.169	0.010	1947-1948
**GAU4502_4**	**CRA23-1948**	**0.0006**	**-**	**0.122**	**0.011**	**1949**
**GAU4502_5**	**CRA23-1949**	**0.0011**	**-**	**0.081**	**0.005**	**1950**
GAU4419/4	*CRA22-1FT-3 1948-1951*	*0.0007*	*0.0002*	-	-	*1949-1952*
**GAU4502_6**	**CRA23-1950**	**0.0005**	**-**	**0.071**	**0.007**	**1951**
**GAU4502_7**	**CRA23-1951**	**0.0036**	**0.0002**	**0.069**	**0.007**	**1952**
GAU4200/6-6	CRA19-2FT-D1 1950-53	0.0048	0.0006	0.109	0.006	1951-1954
**GAU4502_8**	**CRA23-1952**	**0.0059**	**0.0002**	**0.078**	**0.005**	**1953**
**GAU4502_9**	**CRA23-1953**	**0.0088**	**0.0003**	**0.098**	**0.006**	**1954**
GAU4200/6-7	CRA19-2FT-D1 1956-57	0.0139	0.0015	0.161	0.085	1957-1958
GAU4419/5	*CRA22-1FT-3 1954-1959*	*0.0143*	*0.0016*	*0.153*	*0.001*	*1955-1960*
GAU4200/6-8	CRA19-2FT-D1 1958-61	0.0140	0.0015	0.149	0.001	1959-1961
**GAU4502_16**	**CRA23-1960**	**0.0175**	**0.0008**	**0.146**	**0.013**	**1961**
**GAU4502_17**	**CRA23-1961**	**0.0278**	**0.0013**	**0.153**	**0.015**	**1962**
**GAU4502_18**	**CRA23-1962**	**0.0299**	**0.0017**	**0.161**	**0.019**	**1963**
**GAU4502_19**	**CRA23-1963**	**0.0278**	**0.0010**	**0.180**	**0.012**	**1964**
**GAU4502_20**	**CRA23-1964**	**0.0260**	**0.0010**	**0.177**	**0.014**	**1965**
**GAU4502_21**	**CRA23-1965**	**0.0211**	**0.0004**	**0.170**	**0.007**	**1966**
GAU4200/6-9	CRA19-2FT-D1 1964-67	0.0178	0.0019	0.181	0.004	1965-1968
GAU4419/6	CRA22-1FT-3 1964-1969	0.0102	0.0013	0.183	0.002	*1965-1970*
GAU4200/6-10	CRA19-2FT-D1 1970-71	0.0043	0.0005	0.183	0.024	1971-1972
GAU4200/6-11	CRA19-2FT-D1 1972-73	0.0032	0.0004	0.185	0.056	1973-1974
GAU4200/6-12	CRA19-2FT-D1 1976-79	0.0021	0.0004	0.189	0.099	1977-1980
GAU4419/7	CRA22-1FT-3 1976-1980	0.0045	0.0006	0.165	0.002	*1977-1981*
GAU4200/6-13	CRA19-2FT-D1 1982-85	0.0011	0.0002	0.217	0.133	1983-1985
GAU4419/8	CRA22-1FT-3 1982-1987	0.0022	0.0003	0.171	0.002	*1983-1988*
GAU4200/6-14	CRA19-2FT-D1 1988-91	0.0008	0.0002	-		1989-1992
GAU4200/6-15	CRA19-2FT-D1 1994-97	0.0009	0.0002	-		1995-1998
GAU4419/9	CRA22-1FT-3 1996-2001	0.0006	0.0002	-	-	*1997-2002*
